# Non-Hertz-Millis scaling of the antiferromagnetic quantum critical metal via scalable Hybrid Monte Carlo

**DOI:** 10.1038/s41467-023-37686-4

**Published:** 2023-05-03

**Authors:** Peter Lunts, Michael S. Albergo, Michael Lindsey

**Affiliations:** 1 Joint Quantum Institute and Department of Physics, University of Maryland, College Park, MD 20742 USA; 2 Center for Computational Quantum Physics, Flatiron Institute, 162 5th Avenue, New York, NY 10010 USA; 3 Center for Cosmology and Particle Physics, New York University, New York, NY 10003 USA; 4 Courant Institute of Mathematical Sciences, New York University, New York, NY 10012 USA

**Keywords:** Phase transitions and critical phenomena, Magnetic properties and materials

## Abstract

A key component of the phase diagram of many iron-based superconductors and electron-doped cuprates is believed to be a quantum critical point (QCP), delineating the onset of antiferromagnetic spin-density wave order in a quasi-two-dimensional metal. The universality class of this QCP is believed to play a fundamental role in the description of the proximate non-Fermi liquid behavior and superconducting phase. A minimal model for this transition is the O(3) spin-fermion model. Despite many efforts, a definitive characterization of its universal properties is still lacking. Here, we numerically study the O(3) spin-fermion model and extract the scaling exponents and functional form of the static and zero-momentum dynamical spin susceptibility. We do this using a Hybrid Monte Carlo (HMC) algorithm with a novel auto-tuning procedure, which allows us to study unprecedentedly large systems of 80 × 80 sites. We find a strong violation of the Hertz-Millis form, contrary to all previous numerical results. Furthermore, the form that we do observe provides good evidence that the universal scaling is actually governed by the analytically tractable fixed point discovered near perfect “hot-spot’" nesting, even for a larger nesting window. Our predictions can be directly tested with neutron scattering. Additionally, the HMC method we introduce is generic and can be used to study other fermionic models of quantum criticality, where there is a strong need to simulate large systems.

## Abstract (web summary)

The quantum critical point associated with the antiferromagnetic spin-density wave transition occurs in many strongly correlated systems. Here the authors study this quantum critical point in a 2D spin-fermion model using an efficient hybrid Monte Carlo method and make predictions for future experiments.

## Introduction

Quantum critical phenomena play an important role in condensed matter physics^1^. Of particular interest are quantum phase transitions in metals, since they are ubiquitous in strongly correlated materials displaying exotic quantum phenomena, most notably high-temperature superconductivity.

These phase transitions are notoriously difficult to study theoretically^2^. This is due to the presence of an extensive number of gapless fermionic modes on the Fermi surface and the strong coupling between these modes and the transition order parameter. These difficulties render nearly all analytical perturbative approaches uncontrolled. On the numerical side, these difficulties are manifested in a large amount of entanglement and nearly ubiquitous sign problems, making it hard for controlled numerical techniques such as tensor networks and quantum Monte Carlo (QMC) to make progress.

The most common of such phase transitions in Nature is the onset of antiferromagnetic (AF) spin-density wave (SDW) order in a metal. This transition exists in many material classes of interest, such as electron-doped cuprates^3^, iron-based materials^4^, and heavy fermion compounds^5^, in which it is believed to generically be a continuous transition. It is often accompanied by a superconducting ‘dome,’ where the maximal *T*_*c*_ occurs near the putative zero-temperature critical point. This makes the (near-critical) SDW fluctuations a strong candidate for the ‘glue’ of Cooper pairs in those materials.

Due to its importance, the theory of this phase transition has received a considerable amount of attention in the last three decades^6–13^. Very early on it was believed to be well described by Hertz-Millis theory^14,15^, although it was soon realized that, due to the dimensionality, the arguments of Hertz-Millis theory are invalid^6–8^. Crucially, the large-N expansion was shown to fail^9^, which left the theory without a controlled approach until the introduction of the fully local *ϵ*-expansion^10,11^. Using these controlled results as guidance, in ref. ^12^ it was shown that there exists a parameter regime where the theory naturally develops a small control parameter, *w*, which is a ratio of velocities, without the need for any dimensional modification. It is then possible to compute observables perturbatively in *w*, giving the only fully controlled analytical calculation of the universal low-energy data for the unmodified problem. However, the parameter regime, or ‘basin of attraction,’ of this solution could not be determined from the arguments of ref. ^12^. It is therefore not clear whether this solution exists for physically relevant parameter values, or only in a minuscule slice of parameter space. One of the central goals of this work is to answer this question.

In the last decade, the SDW transition in metals has also been studied extensively using numerical techniques. The seminal work of ref. ^16^ introduced a microscopic two-band model of the effective field theory for this transition, which crucially lacks a sign problem. This has led to many studies of this model with Determinantal Quantum Monte Carlo (DQMC)^17–25^, as well as works that have studied other sign-problem-free models of quantum criticality^26–32^.

A recent such DQMC work^21^ focused on the critical scaling of the spin susceptibility, in particular seeking a comparison to the predictions of ref. ^12^ by tuning the UV value of the nesting parameter, *v* (explained in detail in Section “Theoretical analysis near perfect nesting”) close to the value at the fixed point of ref. ^12^, *v* = 0. However, at criticality, the spin susceptibility was actually observed to have a (nearly-perfect) Hertz-Millis form, contradicting the theoretical finding^6–8^ that the Hertz-Millis arguments are not valid in two dimensions. Importantly, the maximal system size studied in ref. ^21^ was *V* = *L* × *L* = 14 × 14. With such a small system size, it is often difficult to convincingly extract long-wavelength behavior of a critical system. Therefore, even though ref. ^21^ is the current state-of-the-art for this problem, it is very desirable to revisit the problem at much larger *L*.

Although DQMC provides a numerically exact and unbiased way to study the properties of these phase transitions, it is severely hindered in its ability to simulate systems with large spatial volume *V* by its computational scaling of at least ~*βV*^3^, where *β* is the inverse temperature (in the case of small fermion density, this can be reduced by exploiting the low-rank structure of the fermionic determinant^33,34^). The true scaling may in fact be worse due to the need to take smaller steps in high dimensions in order to maintain a nonvanishing Metropolis acceptance probability, as well as the presence of ‘critical slowing down,’ discussed further below. Improving the computational scaling with respect to *V* is of great interest in the study of quantum criticality. Indeed, in order to extract scaling properties near a quantum critical point (QCP), it is crucial to be ‘close enough’ to the thermodynamic limit *V* → *∞*. This ‘close enough’ is never possible to determine a priori, and, in principle, due to potential semi-stable fixed points, there is never any reason to expect that it has been reached, unless the observed scaling matches a predicted result.

In this paper, we use a different QMC method to study the microscopic model of ref. ^16^, namely Hybrid Monte Carlo (HMC), which is sometimes referred to as Hamiltonian Monte Carlo. This method is the main numerical tool in the study of lattice quantum chromodynamics (LQCD). Its primary advantage over DQMC is the potential for improved computational scaling with respect to *V*, which we explain in more detail below. HMC has seen a recent revival in condensed matter physics^35–37^. Several works have used it to study the half-filled Hubbard model on various lattices (square, honeycomb, hexagonal)^37–43^, electron-phonon models^37,44^, as well as extended and long-range Hubbard models of graphene^45–49^. Nearly all of these results point to extremely favorable scaling with *V*. However, to the best of our knowledge, HMC has not yet been applied to a model of quantum criticality in the presence of a Fermi surface.

Using our large-scale simulations, we find that the critical theory does in fact strongly deviate from the Hertz-Millis prediction. By tuning the nesting parameter, *v*, closer to the fixed-point value of *v* = 0, we observe a systematic reduction of the dynamical critical exponent *z* below the value of *z* = 2 predicted by Hertz-Millis. The prediction of ref. ^12^ is that *z* → 1^+^ as *v* → 0. Additionally, we find that the momentum dependence of the critical spin susceptibility is O(2)-symmetric at intermediate momenta, as predicted by Hertz-Millis, but at lower momenta the symmetry gets reduced to C_4_, as predicted in ref. ^12^. These two findings provide strong numerical evidence that the criticial point theory is governed by the fixed point of ref. ^12^, even at values of *v* that are appreciable. This is summarized in Table 1. Our predictions for the dynamical spin susceptibility can be directly tested with neutron scattering.

**Table 1:** Summary of results

	*z*(*θ*)	Symmetry	Functional form	
This work	∈ (1.665(29), 1.953(35)) for *θ* ∈ (0. 5^°^, 8^°^)	C_4_	$$\begin{array}{ll}{\chi }^{-1}(\omega ) \sim|\omega {|}^{{{\Delta }}}&z{{\Delta }}\in (1.466(14),1.521(15))\\ {\chi }^{-1}({q}_{x}) \sim|{q}_{x}{|}^{z{{\Delta }}}&{{\Delta }}\in (0.880(12),0.779(12))\\ {\chi }^{-1}({{{{{{{\bf{q}}}}}}}}) \sim|{q}_{x}{|}^{z{{\Delta }}}+|{q}_{y}{|}^{z{{\Delta }}}&{{{{{{{\rm{for}}}}}}}}\,\theta \in (0.{5}^{\circ },{8}^{\circ })\end{array}$$	
Ref. ^12^	*z* → 1^+^ as *θ* → 0	C_4_	$$\begin{array}{ll}{\chi }^{-1}(\omega ) \sim|\omega {|}^{{{\Delta }}}&\\ {\chi }^{-1}({q}_{x}) \sim|{q}_{x}{|}^{z{{\Delta }}}&z{{\Delta }},{{\Delta }}\to {1}^{+}\,{{{{{{{\rm{as}}}}}}}}\,\theta \to 0\\ {\chi }^{-1}({{{{{{{\bf{q}}}}}}}}) \sim \,{{{{{{{\rm{not}}}}}}}}\,{{{{{{{\rm{determined}}}}}}}}&\end{array}$$	
Hertz-Millis	*z* = 2 ∀ *θ*	O(2)	$$\begin{array}{l}{\chi }^{-1}(\omega ) \sim|\omega|\\ {\chi }^{-1}({q}_{x}) \sim {q}_{x}^{2}\\ {\chi }^{-1}({{{{{{{\bf{q}}}}}}}}) \sim {q}_{x}^{2}+{q}_{y}^{2}\end{array}$$	

The main results of this paper, which pertain to the critical spin susceptibility, *χ*(*ω*, **q**). We list its dynamical critical exponent *z*, spatial symmetry, and functional form, as compared to that predicted in ref. ^12^ and by Hertz-Millis theory, all for the varying nesting angle *θ* we study (here we use *θ* instead of the nesting parameter $v=\tan (\theta )$ that we use in the rest of the paper). The forms quoted for ref. ^12^ are neglecting the logarithmic flow of *v*.

In our largest computations we reach system sizes *N*_*τ*_ × *L* × *L* given by *N*_*τ*_ = 200, *L* = 80 and *N*_*τ*_ = 800, *L* = 20, where *N*_*τ*_ is the number of slices in the imaginary time direction. Going to such large scales turns out to be necessary for extracting accurate critical scaling behaviors at the critical point.

Now we give an overview of the computational scaling properties of our approach that allow us to achieve these results. Within the HMC algorithm, the number of ‘integration steps’ per effective sample can enjoy scaling as low as *O*(*d*^1/4^), where *d* = *O*(*βV*) is the dimension of our discretized field to be sampled^50^. This scaling may be worse in the presence of ‘critical slowing down’ for critical models, yielding a number of integration steps per effective sample of potentially $O({\beta }^{1/4+{z}_{1}}{V}^{1/4+{z}_{2}})$, where *z*_1_, *z*_2_ > 0. Even in the presence of criticality, *z*_1_ and *z*_2_ may be reduced via the choice of the ‘metric’ within HMC, as discussed below. For the critical model, our algorithm in fact achieves *z*_2_ ≈ 0 and *z*_1_ ≲ 0.5. Each of the integration steps within HMC requires the solution of linear system of size *O*(*βV*), which constitutes the bottleneck for the algorithm. Our approach for solving the linear system in fact scales linearly with respect to *V* for fixed *β* and permits fast GPU implementation. In conjunction with the scaling *z*_2_ ≈ 0 observed above, this performance yields overall wall clock scaling with exponent approximately 5/4 with respect to *V*. See Section “Numerical Performance” for further details.

In our implementation we in fact develop several augmentations of the basic HMC algorithm. First, we introduce an auto-tuning procedure, which tunes our hyperparameters in an initial warmup phase. This procedure is common practice in statistics and industry applications of HMC^51,52^ but to the best of our knowledge has not yet been fully applied in condensed matter physics. Moreover, relative to such works, our translation-invariant physical setting allows us to tune the aforementioned HMC metric with operations that scale linearly in *βV*, up to log factors.

In the Results Section, we introduce the low-energy effective theory of the SDW transition, both in the continuum and on the lattice, we review the theoretical results near perfect nesting of ref. ^12^ and derive its predictions for the observables that are computed numerically in this work, and we show our main results. In the Methods Section we give a brief review of HMC, followed by a detailed presentation of our implementation and its numerical performance.

## Results

### Continuum effective action

**Fig. 1:**
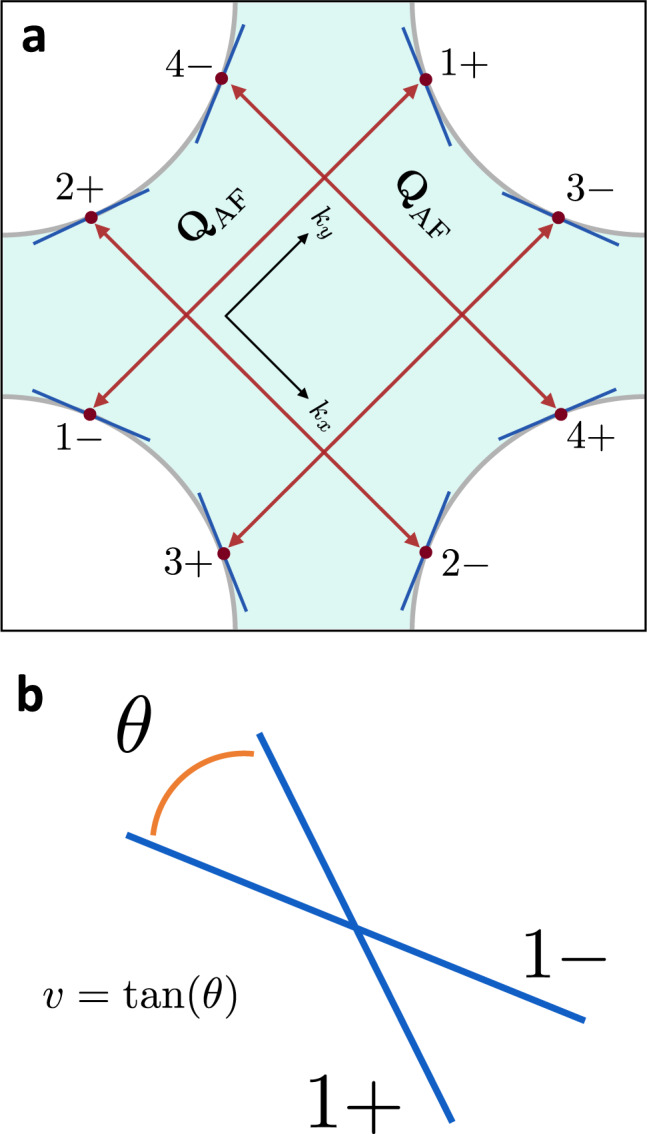
The Fermi surface. **a** The first Brillouin zone of the model in Eq. (1). Note the axes rotated by 45^°^. The shaded region corresponds to the occupied states. The SDW ordering wavevector **Q**_AF_ is denoted by red arrows. The hot spots are the red points connected by **Q**_AF_. At each hot spot, the linearized Fermi surface is shown with a blue line. **b** A close-up of linearized dispersions of the paired hot spots (1, ±). One has been shifted by **Q**_AF_, to show their crossing. The angle between them *θ* is called the `nesting angle', and its tangent $v=\tan (\theta )$ is called the `nesting parameter'.

Our starting point is the theory that describes the low-energy degrees of freedom near a metallic AF SDW quantum critical point in two spatial dimensions. These are the order parameter for the transition, which is the collective spin excitation, and the electrons near points on the Fermi surface called ‘hotspots’ that are connected by the AF ordering wavevector. For concreteness, we consider a single band with C_4_ symmetry and an ordering wavevector equal to (*π*, *π*), as shown in Fig. 1. Generically for such a Fermi surface there are four pairs of coupled hotspots. The Euclidean-time action for this low-energy theory is then given by

1 $${{{{{{{\mathcal{S}}}}}}}}=	 \mathop{\sum }\limits_{n=1}^{4}\mathop{\sum}\limits_{m=\pm }\mathop{\sum}\limits_{\sigma=\uparrow,\downarrow }\mathop{\sum }\limits_{j=1}^{{N}_{f}}T\mathop{\sum}\limits_{{\omega }_{k}}\int\frac{d{{{{{{{\bf{k}}}}}}}}}{{(2\pi )}^{2}}\\ 	 {\psi }_{n,\sigma,j}^{(m)*}(k)\left[i{\omega }_{k}+{e}_{n}^{m}({{{{{{{\bf{k}}}}}}}};v)\right]{\psi }_{n,\sigma,j}^{(m)}(k)\\ 	+T\mathop{\sum}\limits_{{\omega }_{q}}\int\frac{d{{{{{{{\bf{q}}}}}}}}}{{(2\pi )}^{2}}\,\frac{1}{2}\left[\frac{{\omega }_{q}^{2}}{{c}^{2}}+({q}_{x}^{2}+{q}_{y}^{2})+r\right]{{{{{{{\boldsymbol{\phi }}}}}}}}(q)\cdot {{{{{{{\boldsymbol{\phi }}}}}}}}(-q)\\ 	+\frac{g}{\sqrt{{N}_{f}}}\mathop{\sum }\limits_{n=1}^{4}\mathop{\sum}\limits_{\sigma,{\sigma }^{{\prime} }=\uparrow,\downarrow }\mathop{\sum }\limits_{j=1}^{{N}_{f}}\int\,d\tau \mathop{\sum}\limits_{{{{{{{{\bf{r}}}}}}}}}\\ 	 \left[{{{{{{{\boldsymbol{\phi }}}}}}}}\cdot {\psi }_{n,\sigma,j}^{(+)*}{{{{{{{{\boldsymbol{\tau }}}}}}}}}_{\sigma,{\sigma }^{{\prime} }}{\psi }_{n,{\sigma }^{{\prime} },j}^{(-)}+{{{{{{{\rm{h.c.}}}}}}}}\right]+\frac{u}{4}\int\,d\tau \mathop{\sum}\limits_{{{{{{{{\bf{r}}}}}}}}}{({{{{{{{\boldsymbol{\phi }}}}}}}}\cdot {{{{{{{\boldsymbol{\phi }}}}}}}})}^{2}.$$

In Eq. (1), *k* = (*ω*_*k*_, **k**) consists of the fermionic Matsubara frequency and the two-dimensional momentum **k** = (*k*_*x*_, *k*_*y*_). *T* is the temperature. The ${\psi }_{n,\sigma }^{(m)}$ correspond to electrons at the hot spots labeled by *n* ∈ {1, 2, 3, 4} and *m* ∈ {+, −} (cf. Fig. 1), and spin *σ* ∈ {*↑*, *↓*}. The axes are chosen as follows: ${\hat{k}}_{x}$ is in the direction from hot spot (2, +) to (2, −) and ${\hat{k}}_{y}$ is in the direction from hot spot (1, −) to (1, +). Given this choice of axes, the ordering wave vector connecting the paired hot spots is ${{{{{{{{\bf{Q}}}}}}}}}_{{{{{{{{\rm{AF}}}}}}}}}=(\pm \sqrt{2}\pi {\hat{k}}_{x},\pm \sqrt{2}\pi {\hat{k}}_{y})$ up to the reciprocal lattice vectors $\sqrt{2}\pi ({\hat{k}}_{x}\pm {\hat{k}}_{y})$. See Fig. 1 for details. The linearized electron dispersions are given by ${e}_{1}^{\pm }({{{{{{{\bf{k}}}}}}}};v)=-{e}_{3}^{\pm }({{{{{{{\bf{k}}}}}}}};v)=v{k}_{x}\pm {k}_{y}$, ${e}_{2}^{\pm }({{{{{{{\bf{k}}}}}}}};v)=-{e}_{4}^{\pm }({{{{{{{\bf{k}}}}}}}};v)= \mp {k}_{x}+v{k}_{y}$, where the momentum **k** is measured relative to each hot spot. For any *v* ≠ 0, the curvature of the Fermi surface (${{{{{{{\mathcal{O}}}}}}}}({k}^{2})$ terms) can be ignored, since the linearized dispersions at the coupled hostpots are not parallel to each other, and therefore the problem is still fully two-dimensional. The component of the Fermi velocity along **Q**_AF_ has been set to one by rescaling **k**. *v* is the component of Fermi velocity that is perpendicular to **Q**_AF_. It controls the degree of nesting between coupled hot spots and can be written as $v=\tan \theta$, where *θ* is the nesting angle (c.f. Fig. 1). ***ϕ***(*q*) is the three-component boson field that describes the AFM collective mode in the fundamental representation of O(3), with frequency *ω*_*q*_ and momentum **q**. Note that while the collective spin is centered at **Q**_AF_, ***ϕ*** is centered at zero, since **Q**_AF_ is already incorporated into the hotspot label. *c* is the boson velocity, and *u* is its quartic interaction. *r* is the squared mass of the boson, as is tuned drive the boson to criticality. *g* is the Yukawa coupling between the boson and the electrons, which scatters the electrons between hot spot pairs via the spin-spin interaction. These couplings represent all the relevant and marginal terms obeying the symmetries of the problem. ***τ*** consists of the three generators of the *SU*(2) group. We have also generalized the action from one to *N*_*f*_ fermion flavors. This generalization was used in the large *N*_*f*_ expansion in previous renormalization group studies^7,9^, and we use this general form in subsequent sections.

### Sign-problem-free UV completion

The action in Eq. (1) captures the universal low-energy properties of the system associated with the divergent correlation length near the phase transition. Since the electrons far away from the hotspots do not enter into the theory, the precise shape of the Fermi surface beyond the momentum cutoffs does not play a role in the critical phenomena of this theory (the complete validity of this has recently been called into question in Ref. ^53^). Therefore, we can change the band structure while ensuring that the action of Eq. (1) is not modified. One such UV completion was given in ref. ^16^ and has the real-space action of

2 $${{{{{{{\mathcal{S}}}}}}}}=	 \int\,d\tau \mathop{\sum}\limits_{\sigma=\uparrow,\downarrow }\mathop{\sum}\limits_{\alpha=x,y}\mathop{\sum }\limits_{j=1}^{{N}_{f}}\mathop{\sum}\limits_{{{{{{{{\boldsymbol{r}}}}}}}},{{{{{{{{\boldsymbol{r}}}}}}}}}^{{\prime} }}\\ 	 {\psi }_{\alpha,\sigma,j,{{{{{{{\boldsymbol{r}}}}}}}}}^{*}\left[({\partial }_{\tau }-\mu ){\delta }_{{{{{{{{\boldsymbol{r}}}}}}}},{{{{{{{{\boldsymbol{r}}}}}}}}}^{{\prime} }}-{t}_{\alpha,{{{{{{{\boldsymbol{r}}}}}}}},{{{{{{{{\boldsymbol{r}}}}}}}}}^{{\prime} }}\right]{\psi }_{\alpha,\sigma,j,{{{{{{{{\boldsymbol{r}}}}}}}}}^{{\prime} }}\\ 	 \int\,d\tau \mathop{\sum}\limits_{{{{{{{{\boldsymbol{r}}}}}}}}}\left[\frac{1}{{c}^{2}}{({\partial }_{\tau }{{{{{{{{\boldsymbol{\phi }}}}}}}}}_{{{{{{{{\boldsymbol{r}}}}}}}}})}^{2}+\frac{1}{2}{(\nabla {{{{{{{{\boldsymbol{\phi }}}}}}}}}_{{{{{{{{\boldsymbol{r}}}}}}}}})}^{2}+\frac{r}{2}{({{{{{{{{\boldsymbol{\phi }}}}}}}}}_{{{{{{{{\boldsymbol{r}}}}}}}}})}^{2}+\frac{u}{4}{({{{{{{{{\boldsymbol{\phi }}}}}}}}}_{{{{{{{{\boldsymbol{r}}}}}}}}})}^{4}\right]\\ 	+\frac{g}{\sqrt{{N}_{f}}}\mathop{\sum}\limits_{\sigma,{\sigma }^{{\prime} }=\uparrow,\downarrow }\mathop{\sum }\limits_{j=1}^{{N}_{f}}\int\,d\tau \mathop{\sum}\limits_{{{{{{{{\boldsymbol{r}}}}}}}}}{e}^{i{{{{{{{{\boldsymbol{Q}}}}}}}}}_{{{{{{{{\rm{AF}}}}}}}}}\cdot {{{{{{{\boldsymbol{r}}}}}}}}}\\ 	 {{{{{{{{\boldsymbol{\phi }}}}}}}}}_{{{{{{{{\boldsymbol{r}}}}}}}}}\cdot \left[{\psi }_{x,\sigma,{{{{{{{\boldsymbol{r}}}}}}}}}^{*}\ {{{{{{{{\boldsymbol{\tau }}}}}}}}}_{\sigma,{\sigma }^{{\prime} }}\ {\psi }_{y,{\sigma }^{{\prime} },{{{{{{{\boldsymbol{r}}}}}}}}}+{{{{{{{\rm{h.c.}}}}}}}}\right].$$

Here, the number of bands has been doubled (*α* = *x*, *y* represents a band index). The boson now scatters electrons between the two bands. The band-dependent hopping amplitude ${t}_{\alpha,{{{{{{{\boldsymbol{r}}}}}}}},{{{{{{{{\boldsymbol{r}}}}}}}}}^{{\prime} }}$ still respects the C_4_ symmetry of the lattice, provided that the bands are also interchanged. The location of the hotspots and the dispersion linearized about them is unchanged from the one-band model. Note that the axes of this model are rotated 45^°^ relative to the model of Eq. (1). The advantage of this model is that it now contains an inter-band anti-unitary symmetry that guarantees the positivity of the fermionic determinant^16^ and makes it amenable to sign-problem-free Monte Carlo simulations^17–21,24,25^.

**Fig. 2:**
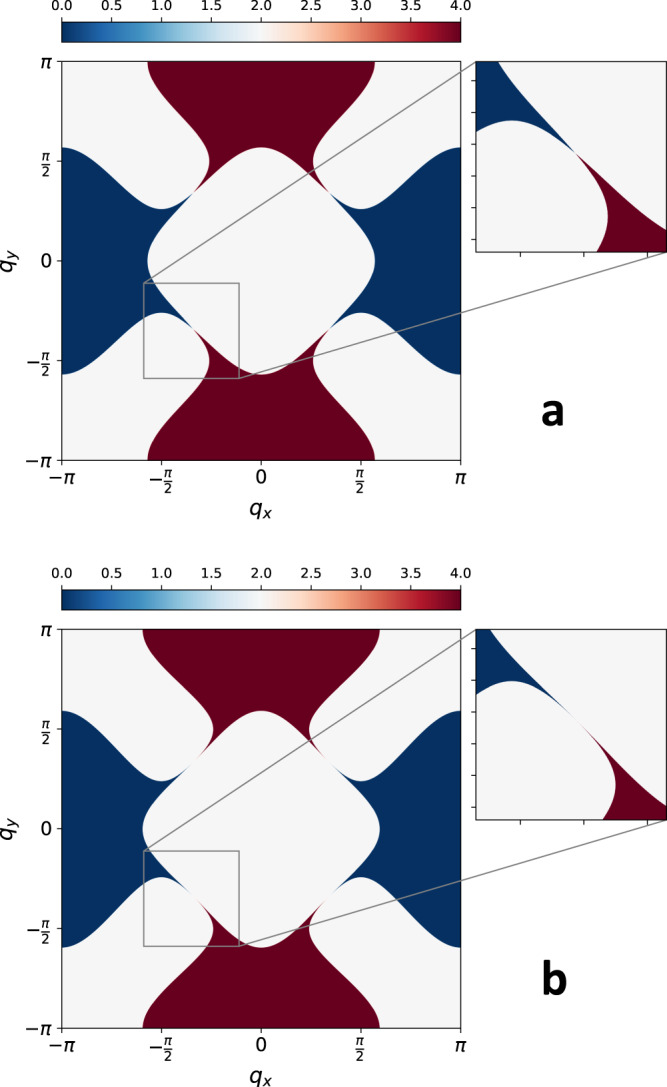
Occupation number. The total occupation number ${n}_{{{{{{{{\boldsymbol{q}}}}}}}}}={\sum }_{\alpha,\sigma }\langle {\psi }_{\alpha,\sigma,{{{{{{{\boldsymbol{q}}}}}}}}}^{{{{\dagger}}} }{\psi }_{\alpha,\sigma,{{{{{{{\boldsymbol{q}}}}}}}}}\rangle$ of one flavor of fermions for the free theory, summed over spin and band degrees of freedom. Here, we have incorporated the phase shift ${e}^{i{{{{{{{{\boldsymbol{Q}}}}}}}}}_{{{{{{{{\rm{AF}}}}}}}}}\cdot {{{{{{{\boldsymbol{r}}}}}}}}}$ into the *α* = *y* band. The nesting values shown are (**a**) *v* = *v*_1_ and (**b**) *v* = *v*_5_, which are the two extremes we study. Note that the Fermi surface at the hot-spots is actually connected, but due to the very small nesting parameter, that is hard to show graphically.

We study the lattice model defined in Eq. (2) using HMC. Our method is described in detail in Section “Methods”. As explained there, in order for our algorithm to function, we must work with an even *N*_*f*_, and we choose *N*_*f*_ = 2. Unless stated otherwise, we fix the parameter values to be $u=0,\,g=0.7\,\sqrt{2}\,\approx \,1,\,c=3$. We study five different Fermi surfaces, with the following values of the nesting parameter: *v*_1_ ≈ 0.149, *v*_2_ ≈ 0.072, *v*_3_ ≈ 0.036, *v*_4_ ≈ 0.018, *v*_5_ ≈ 0.0092, corresponding to nesting angles of *θ*_1_ ≈ 8. 5^°^, *θ*_2_ ≈ 4.13^°^, *θ*_3_ ≈ 2.05^°^, *θ*_4_ ≈ 1.03^°^, *θ*_5_ ≈ 0.53^°^, respectively. We illustrate the Fermi surface corresponding to *v* = *v*_1_ and *v* = *v*_5_ in Fig. 2. The non-zero hopping amplitudes and chemical potentials corresponding to each are *t*_*h*,*x*_ = *t*_*v*,*y*_ = 1 and

3 $${\tilde{t}}_{v,x}^{(1)}=-{\tilde{t}}_{h,y}^{(1)}=0.45,\quad {\mu }_{x}^{(1)}=-{\mu }_{y}^{(1)}=-0.47$$

4 $${\tilde{t}}_{v,x}^{(2)}=-{\tilde{t}}_{h,y}^{(2)}=0.48,\quad {\mu }_{x}^{(2)}=-{\mu }_{y}^{(2)}=-0.46$$

5 $${\tilde{t}}_{v,x}^{(3)}=-{\tilde{t}}_{h,y}^{(3)}=0.498,\quad {\mu }_{x}^{(3)}=-{\mu }_{y}^{(3)}=-0.44$$

6 $${\tilde{t}}_{v,x}^{(4)}=-{\tilde{t}}_{h,y}^{(4)}=0.505,\quad {\mu }_{x}^{(4)}=-{\mu }_{y}^{(4)}=-0.44$$

7 $${\tilde{t}}_{v,x}^{(5)}=-{\tilde{t}}_{h,y}^{(5)}=0.5085,\quad {\mu }_{x}^{(5)}=-{\mu }_{y}^{(5)}=-0.44.$$

Here, *t*_*h*,*α*_, *t*_*v*,*α*_ denote the nearest-neighbor hopping amplitudes in the *x* and *y* directions, respectively, which are the same for all nesting parameter values; ${\tilde{t}}_{h,\alpha }^{(i)},\,{\tilde{t}}_{v,\alpha }^{(i)}$ denote the next-nearest-neighbor hopping amplitudes in the *x* and *y* directions, respectively, for nesting parameter value *i*; ${\mu }_{\alpha }^{(i)}$ are the chemical potentials for the nesting parameter value *i*.

The reason for choosing these specific parameter values is to make contact with ref. ^21^, where the authors in turn tried to make contact with ref. ^12^. Of course, it is important to scan the values of *u*, *g*, *c* to check the stability of our results and look for new behavior. However, such a detailed study is beyond the scope of the present work, and is left for future studies.

### Theoretical analysis near perfect nesting

Although in general the theory in Eq. (1) cannot be understood analytically using a controlled approach, there exists a parameter regime where a controlled solution in the IR can be obtained^12^. This parameter regime is primarily characterized by a small nesting parameter *v* ≪ 1 and an effective coupling of intermediate strength, leading to strong correlations. We start by reviewing this IR fixed point of refs. ^11,12,54^.

**Fig. 3:**
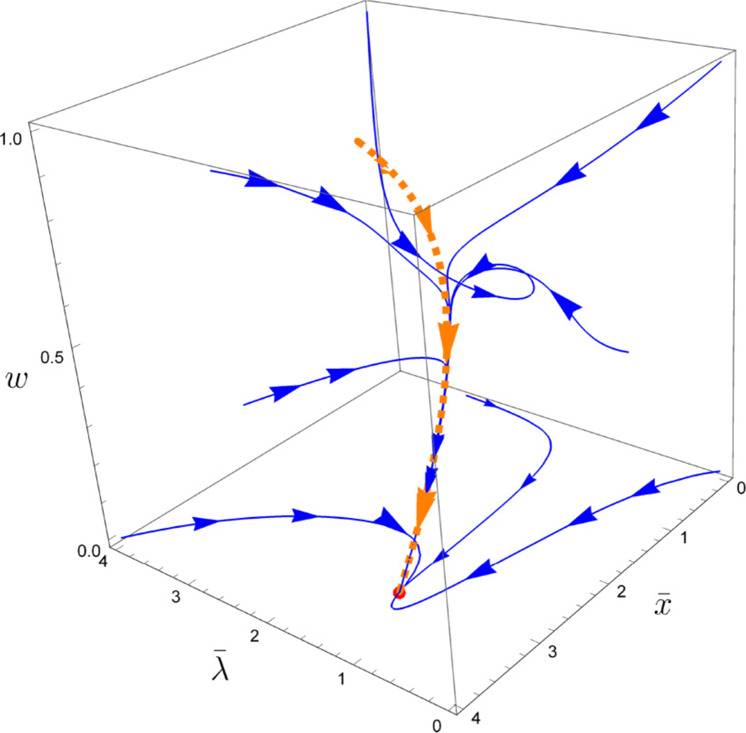
Renormalization group flow. RG flow of the theory in Eq. (1), computed using the epsilon expansion, reproduced from ref. ^11^. The axes are *w*, $\bar{x}=x/10$, $\bar{\lambda }=10\lambda$. Initially, there is a fast flow (blue lines) to a fixed one-dimensional manifold (dashed orange line). The flow along this manifold is slow, and towards *w* = 0. The flow of *κ* is excluded, since its flow towards zero is the fastest.

At criticality, the UV the theory is best described in terms of the ratios $\lambda \equiv \frac{{g}^{2}{c}^{2}}{v},\,x\equiv \frac{{g}^{2}}{c}$, *κ* ≡ *u* *c*^2^, and $w=\frac{v}{c}$. (We note that compared to refs. ^11,12,54^, the action of Eq. (1) has ***ϕ*** → ***ϕ***/*c*). The renormalization group flow of the UV theory to the IR fixed point is two-fold and is shown in Fig. 3, which is reproduced from ref. ^11^. Initially, there is a fast (algebraically in the running energy scale *μ*) flow of *λ* and *x* to *w*-dependent ${{{{{{{\mathcal{O}}}}}}}}(1)$ values and *κ* to zero. Once the first step of the flow is complete, the couplings will keep flowing along the one-dimensional manifold defined by the fixed point values *λ*, *x*, *κ*. This one-dimensional manifold defines the only remaining free coupling of the theory. We note that since $\lambda \sim {{{{{{{\mathcal{O}}}}}}}}(1)$ is the effective coupling of the UV theory, this manifold represents the theory in the strongly correlated regime. Crucially, due to all the coupling inter-dependencies, along the manifold *w* becomes a function of *v* only. We can choose to parametrize the manifold by *w*(*v*). This choice is useful, since it turns out that theory on the manifold has a perturbation theory that is organized in powers of *w*(*v*) (along with powers of $\log (w)$). However, since *w*(*v*) is only a function of *v*, such that *w*(*v*) → 0 with *v* → 0, perturbation theory can also be done in *v* itself.

Finally, once the theory sits on the one-dimensional manifold, the remaining flow is towards *w*(*v*) → 0. However, unlike the first part of the flow, the flow of *w*(*v*) in this second part happens at a rate that is sub-logarithmic in *μ*. Therefore, for all practical purposes we can take *w*(*v*) (and therefore *v*) to be fixed (scale independent) on this manifold. An important point is that due to the initial fast flow, the value of *v* on the manifold is different than the bare (UV) value *v*_*B*_. However, given that the bare parameters of the theory are tuned to minimize the length of this initial flow, we assume that *v* ≈ *v*_*B*_ in the analysis of Section “Results”.

If the value of *w*(*v*) is small enough, the theory can be studied using perturbation theory in *w*(*v*) to a finite order. Focusing on the critical spin susceptibility *χ*(*ω*, ***q***), its leading order in *w*(*v*) behavior is given by (here we use the axes of Eq. (1))

8 $${\chi }^{-1}(\omega,\,{{{{{{{\boldsymbol{q}}}}}}}})=\left|\omega \right |+c(w(v))\,(\left|{q}_{x}\right |+\left|{q}_{y}\right|).$$

Here, *c*(*w*(*v*)) is a new emergent boson velocity, not related to the bare value *c*, and dependent on the only coupling in the theory. It has its own expansion in powers of *w*(*v*)^12^. This form breaks the O(2) spatial symmetry (a $\left|{{{{{{{\boldsymbol{q}}}}}}}}\right|$ dependence) of the spin susceptibility down to the C_4_ symmetry of the Fermi surface. This is a key prediction of the small *v* fixed point theory^12^. Equation (8) can be thought of as the tree-level susceptibility at the fixed point, and perturbations to it are computed in powers of *w*(*v*). The deviations of the scaling are encoded in the dynamical critical exponent *z* and the anomalous boson dimension *η*_*ϕ*_, which were computed to leading order in ref. ^12^,

9 $$z=1+\frac{3}{4\pi {N}_{f}}w(v)$$

10 $${\eta }_{\phi }=\frac{1}{2\pi {N}_{f}}w(v)\log \left(\frac{1}{w(v)}\right).$$

Reference^12^ also computed the dependence of *w*(*v*) on *v* to lowest order in *v*,

11 $$w=4\sqrt{{N}_{f}}\sqrt{\frac{v}{\log (1/v)}}.$$

Together these relations give the leading contributions to *z*, *η*_*ϕ*_ for small *v*.

In order to make a connection between the leading-order pertubation theory in *w*(*v*) results of ref. ^12^ and the theory of Eq. (2) at finite *v*, we need to use the non-perturbative renormalization group (RG) equation. This equation for *χ*(*ω*, ***q***) at criticality (*r* = *r*_*c*_) is given by

12 $$\begin{array}{ll}&\left[{{\Delta }}({\eta }_{\phi },\,z)+\omega \frac{\partial }{\partial \omega }+\frac{1}{z}{{{{{{{\boldsymbol{q}}}}}}}}\cdot \frac{\partial }{\partial {{{{{{{\boldsymbol{q}}}}}}}}}+T\frac{\partial }{\partial T}\right.\\ &\left.+\frac{1}{z}\left(\frac{1}{L}\right)\frac{\partial }{\partial \left(\frac{1}{L}\right)}\right]\chi (\omega,{{{{{{{\boldsymbol{\,q}}}}}}}};T,\,L)=0,\end{array}$$

where ${{\Delta }}({\eta }_{\phi },\,z)=\frac{1-2{\eta }_{\phi }-(z-1)}{z}$. Here, we have used the fact that *v* ≈ *v*_*B*_ is approximated as being constant as a function of scale (otherwise its beta function would enter in Eq. (12)). Since at criticality the correlation length is absent from Eq. (12), we have included *T*, *L*^−1^ as relevant energy scales. We note that for a scaling theory different from that of ref. ^12^, the RG equation would be the same, with the only modification being the form of Δ. The only requirement is that the flow of the couplings can be ignored, which is a good approximation when the couplings flow at most logarithmically in the running scale.

We study systems of fixed size and temperature. We focus on two limits of Eq. (12). In the first limit, ***q*** = 0, the solution is given by

13 $$\chi (\omega )={\left|\omega \right|}^{-{{\Delta }}({\eta }_{\phi },z)}f\left(\omega /T,\,\omega {L}^{z}\right),$$

where *f* is some non-universal crossover function. Here, *T* and 1/*L* act as IR cutoffs to the critical scaling. In other words, there is some effective IR frequency cutoff given roughly by ${\omega }_{{{{{{{{\rm{IR}}}}}}}}} \sim \max (\frac{2\pi }{\beta },\,{(\frac{2\pi }{L})}^{z})$, and the function $f\left(\omega /T,\,\omega {L}^{z}\right)$ is a finite constant for *ω* ≫ *ω*_IR_. On the other hand, the scaling equation itself only holds below some non-universal UV cutoff Λ_*ω*_. Therefore, the pure algebraic scaling of $\chi (\omega,\,{{{{{{{\boldsymbol{q}}}}}}}}=0) \sim {\left|\omega \right|}^{-{{\Delta }}({\eta }_{\phi },z)}$ can be observed in an intermediate frequency window, *ω*_IR_ ≪ *ω* ≪ Λ_*ω*_.

Now we turn to the case of *ω* = 0. For simplicity, we first also set *q*_*y*_ = 0. Analogously to *χ*(*ω*), *χ*(*q*_*x*_) is given by

14 $$\chi ({q}_{x})={\left|{q}_{x}\right|}^{-z{{\Delta }}({\eta }_{\phi },z)}\ g({q}_{x}/{T}^{1/z},\,{q}_{x}L).$$

Here, *g* is another non-universal crossover function. The critical scaling $\chi ({q}_{x}) \sim {\left|{q}_{x}\right|}^{-z{{\Delta }}({\eta }_{\phi },z)}$ holds in the intermediate region of *q*_IR_ ≪ *q*_*x*_ ≪ Λ_*q*_, where ${q}_{{{{{{{{\rm{IR}}}}}}}}}=\max ({(\frac{2\pi }{\beta })}^{1/z},\,\frac{2\pi }{L})$ and Λ_*q*_ are IR and UV momentum cutoffs, respectively. Once the intermediate-scale algebraic scaling of both *χ*(*ω*) and *χ*(*q*_*x*_) are known, *z* is computed directly from their ratio: *z*Δ(*η*_*ϕ*_, *z*)/Δ(*η*_*ϕ*_, *z*).

When we turn on *q*_*y*_, the RG equation does not say anything about the functional form of the dependence of *χ*(***q***) on *q*_*x*_ and *q*_*y*_, which must be deduced by other means. All that is known from the tree-level form of Eq. (8) is that the spatial dependence must be C_4_-symmetric. Jumping ahead to Section “Results”, the Monte Carlo data suggests the form

15 $${\chi }^{-1}({{{{{{{\boldsymbol{q}}}}}}}})=	 ({\left|{q}_{x}\right|}^{z{{\Delta }}({\eta }_{\phi },z)}+{\left|{q}_{y}\right|}^{z{{\Delta }}({\eta }_{\phi },z)})\ \\ 	 g((\left|{q}_{x}\right |+\left|{q}_{y}\right|)/{T}^{1/z},\,(\left|{q}_{x}\right |+\left|{q}_{y}\right|)\,L),$$

which we take as our conjecture.

As as aside, we note that this way of extracting critical exponents from a system at fixed *L*, *β* is not the usual one of finite size scaling. Although the latter is more systematic, it would require the simultaneous scaling of *L* and *β* ∝ *L*^*z*^. Since we do not know what *z* is a priori, this requires an additional scan over its potential values. Also, the *z* value we find later on is always *z* > 1.6, which implies the need for a large *β* in the finite size scaling. Both of these are very computationally expensive, and we therefore opt for the present method.

### Numerical results

**Fig. 4:**
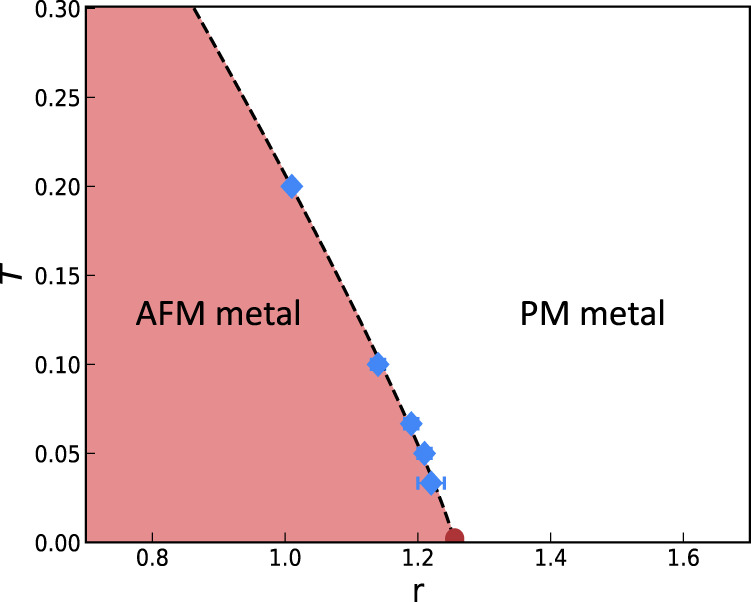
Phase diagram in *T* and *r*. The red (white) region is a metal with AFM (no magnetic) order. The blue points denote the intersection of curves of the Binder cumulant for different system sizes at fixed temperatures. The error bars denote the finite resolution in *r* that we have in those intersection points. The phase diagram for all nesting parameters studied is the same up to our resolution. Superconductivity is absent for all the parameters we study.

The phase diagram of the theory in Eq. (2) as a function of *r* and *T* is shown in Fig. 4.

At large values of *r* the system is a paramagnetic (PM) metal. As a function of decreasing *r*, for any finite *T* (below some large temperature) there is a sharp crossover to a SDW metal. This crossover will not become a true second-order phase transition in the thermodynamic limit, since the dimensionality and symmetry of the order parameter prevent the system from ordering due to the Mermin-Wagner theorem. However, if we infinitesimally couple stacks of this two-dimensional system (as is the situation in many relevant experiments), order will be stabilized and the transition will become a true one. We can identify the sharp location of this potential second-order transition, *r*_*c*_(*T*), by studying finite-size scaling diagnostics, such as the Binder cumulant (c.f. Supplementary Note 1 for details). The line of these transition points terminates at *T* = 0 and *r* = *r*_*c*_, which is the quantum critical point. This is the familiar picture of quantum criticality.

For all nesting parameters we see no superconductivity down to the lowest measured temperatures. This is consistent with the arguments outlined in ref. ^21^ that the superconducting transition temperature *T*_*c*_ of the spin-fermion model is suppressed with decreasing *v* as ${T}_{c} \sim {g}^{2}\sin (\arctan (v))$.

**Fig. 5:**
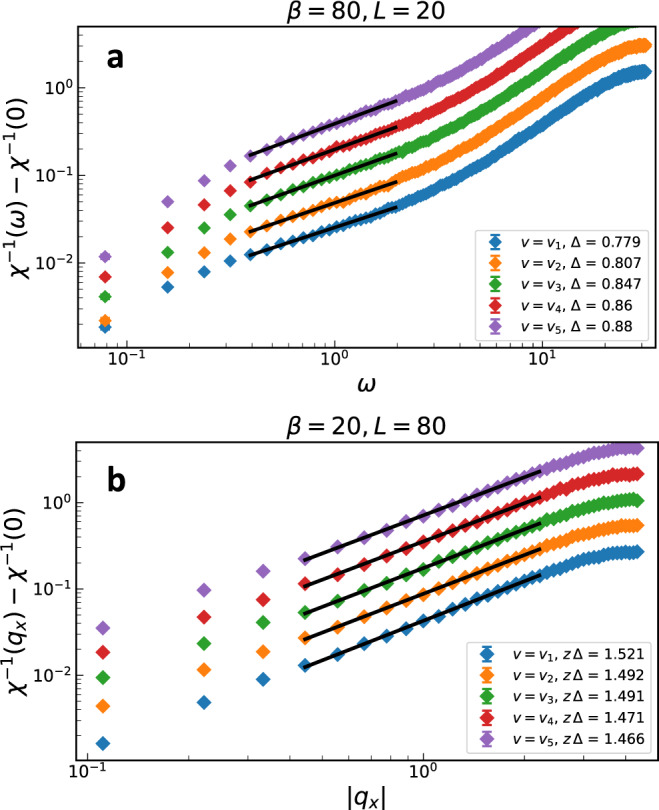
Scaling of critical spin susceptibility. The (**a**) dynamic and (**b**) static spin susceptibility, shown on log-log plots for all nesting parameters *v*_*i*_ studied. The error bars are given by the one sigma statistical uncertainties from the stochastic calculation. The different curves are shifted relative to each other for visual clarity. We show only the largest (**a**) *β* and (b) *L* values that we simulated in order to extract the intermediate *ω* and *q*_*x*_ regimes of power-law scaling. For each curve, we choose the regions that look the most straight by eye (if there are two such regions, as in *χ*^−1^(*ω*), we choose the one with smaller *ω* or *q*_*x*_). Choosing slightly different boundaries leads to slightly different exponents, but the trend with *v* is always the same.

**Fig. 6:**
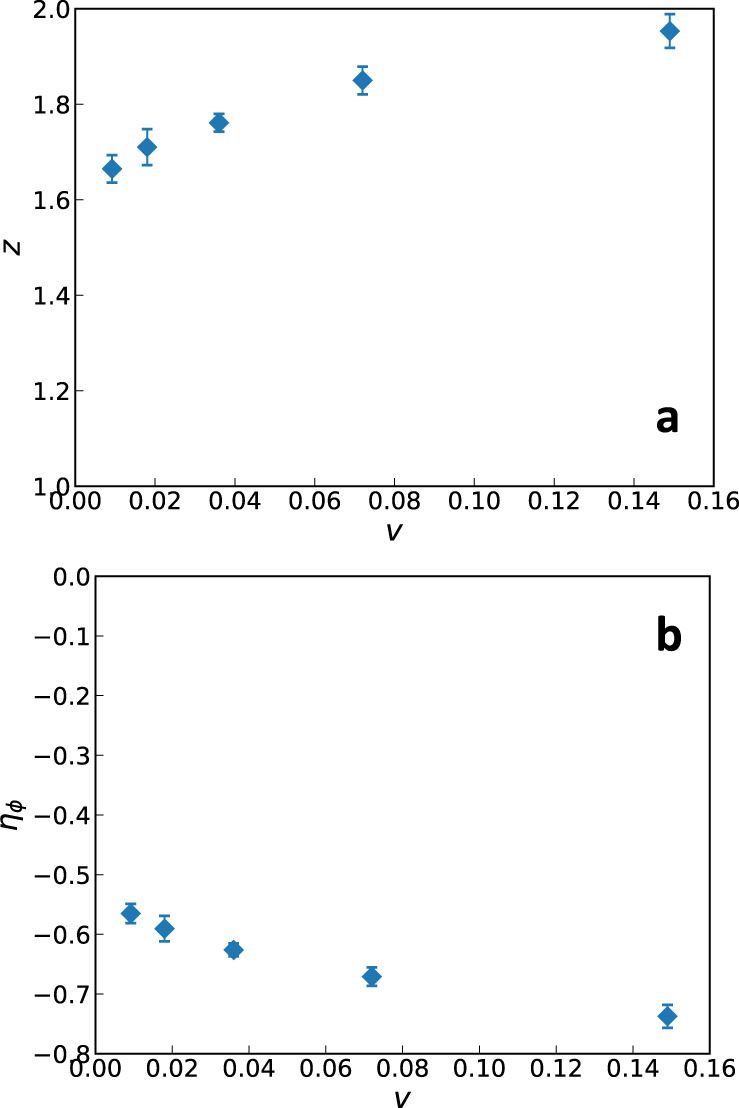
Critical exponents. The (**a**) dynamical critical exponent *z* and (**b**) the anomalous boson dimension *η*_*ϕ*_ as a function of *v*. The error bars are computed using a bootstrap method on the fitted data, allowing for duplicates (c.f. Supplementary Note 1).

To study the critical scaling we tune *r* = *r*_*c*_ and vary *β* ≡ 1/*T*. Following Section “Theoretical analysis near perfect nesting”, we look at the dependence of the spin susceptibility on frequency and momentum, *χ*(*ω*), *χ*(*q*_*x*_), *χ*(***q***). Here, we use the axes of Eq. (1), in order to compare to Section “Theoretical analysis near perfect nesting”. From the scaling of *χ*(*ω*) and *χ*(*q*_*x*_) in the intermediate scaling regions we obtain the exponents Δ(*η*_*ϕ*_, *z*) and *z* Δ(*η*_*ϕ*_, *z*), as illustrated in Fig. 5. From Δ(*η*_*ϕ*_, *z*) and *z* Δ(*η*_*ϕ*_, *z*) we determine *z* and *η*_*ϕ*_, which are plotted in Fig. 6 as functions of *v*. An important point is that the intermediate regime of power-law scaling in Fig. 5 is chosen by eye, and different choices yield slightly different forms of *z*(*v*) and *η*_*ϕ*_(*v*). The specific choice we use in Fig. 5 is described in Supplementary Note 1, along with how we compute the corresponding error bars. However, crucially, for all (reasonable) choices of the scaling region boundaries, *z*(*v*) monotonically decreases with decreasing *v*, starting from a value slightly less than *z* = 2 (which is the Hertz-Millis prediction) for *v*_1_. Similarly, *η*_*ϕ*_(*v*) monotonically increases from *η*_*ϕ*_ ≈ − 0.75 with decreasing *v*.

**Fig. 7:**
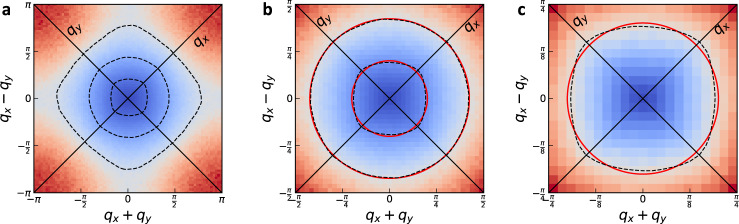
Density plots of  the static critical spin susceptibility. All are for *v* = *v*_5_, *β* = 20 and *L* = 80. We are interested in showing the equal-density contours (we do not show a color bar as it is irrelevant for this purpose). The three plots are different levels of zooming in to ***q*** = 0. In **a** we show the entire Brillouin zone, along with three dashed-line equal-density contours (passed through a Gaussian filter for smoothness), at large, intermediate, and small momenta. We can see that the largest contour has a C_4_-symmetric form, due to the lattice. In **b** we zoom in to focus on the inner two contours, which we overlay with two red circles at radii 0.63, 1.33, in order to illustrate their symmetry. The larger circle overlays with the contour extremely well, indicating an O(2) symmetry at those momenta. The smaller circle overlays with its contour poorly, since that contour is much more square-like, indicating that at smaller momenta the symmetry is again C_4_. To show this more convincingly, in **c** we zoom in further to enlarge this smallest contour.

In addition to the dependence of *z*(*v*) and *η*_*ϕ*_(*v*) on *v*, we determine the spatial symmetry of *χ*(***q***) and its functional form. In Fig. 7 we show density plots of *χ*^−1^(***q***) for *v* = *v*_5_. We plot contours at various values. We can see that at large momenta, the symmetry of the contours is that of the lattice, C_4_. At intermediate momenta, the contours are circles, indicating an O_2_ symmetry, indicating that those momenta are small enough that $\cos ({k}_{x})+\cos ({k}_{y})\,\approx \,2-{{{{{{{{\boldsymbol{k}}}}}}}}}^{2}/2$ is a good approximation. However, at smaller momenta the contours transform again to a C_4_-symmetric form, with the maxima/minima now rotated by 45^°^. In Supplementary Note 1, we show more density plots at smaller *L*, in order to illustrate that this effect gets more pronounced as *L* increases and is therefore a true long-wavelength effect present in the thermodynamic limit.

**Fig. 8:**
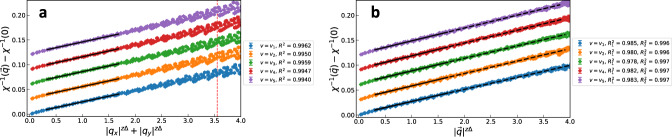
Scaling form of static critical spin susceptibility. *χ*^−1^(***q***) − *χ*^−1^(0) plotted against (**a**) ${\left|{q}_{x}\right|}^{z{{\Delta }}}+{\left|{q}_{y}\right|}^{z{{\Delta }}}$ and (**b**) ${\left|{{{{{{{\boldsymbol{q}}}}}}}}\right|}^{z{{\Delta }}}$. Both plots are for *β* = 20 and *L* = 80. The error bars are again the one sigma uncertainties. The curves for different *v*_*i*_ are shifted to separate them for visual clarity. The initial region, up to *q*^*z*Δ^ ~ 1.7 is fit with both forms. The C_4_ is much better in this region; see the solid lines in **a** and **b**, and compare the coefficients of determination *R*^2^ from **a** with ${R}_{1}^{2}$ from **b**. However, over larger momenta (up to *q*^*z*Δ^ ~ 3.6) the O(2) fit it much better; see the dashed line as well as ${R}_{2}^{2}$ in **b**.

It is not possible to provably determine the exact functional form, as there are many forms that obey the C_4_ symmetry. However, ${\left|{q}_{x}\right|}^{z{{\Delta }}}+{\left|{q}_{y}\right|}^{z{{\Delta }}}$ seems to fit very well. We illustrate this in Fig. 8, along with a comparison to ${\left|{{{{{{{\boldsymbol{q}}}}}}}}\right|}^{z{{\Delta }}}$. We therefore conjecture that ${\chi }^{-1}({{{{{{{\boldsymbol{q}}}}}}}}) \sim {\left|{q}_{x}\right|}^{z{{\Delta }}}+{\left|{q}_{y}\right|}^{z{{\Delta }}}$ is the correct long-wavelength form.

These two features of the boson susceptibility—i.e., the monotonic forms of *z*(*v*), *η*_*ϕ*_(*v*) and the symmetry reduction of O(2) → C_4_ in the long-wavelength limit—provide strong numerical evidence in favor of the critical scaling at the SDW QCP being governed by the theory of ref. ^12^ for all the nesting values we study.

Despite the similarities, there are two parts of the data that are seemingly in contrast to the predictions of ref. ^12^. The first is the fact that *η*_*ϕ*_ is negative, whereas Eq. (10) predicts that as *η*_*ϕ*_ → 0^+^ as *v* → 0. However, Eq. (10) is only the leading order correction, and it particular, in obtaining it, terms of ${{{{{{{\mathcal{O}}}}}}}}(w(v))$ were ignored in ref. ^12^. Therefore, it is perfectly possible that the actual leading-order coefficient behaves as ${\eta }_{\phi } \sim w(v)(\log (1/w(v))-a)$, where *a* > 0 is a constant, which would lead to a sign-change in the leading-order behavior when 1 ≫ *w*(*v*) > *e*^−*a*^. On the other hand, the leading term of *z* from Eq. (9) does not suffer from a similar problem, and its sign can be trusted at small *w*(*v*).

The second part of the data that is seemingly in contrast to the predictions of ref. ^12^ is that *z*(*v*) and *η*_*ϕ*_(*v*) do not convincingly exhibit the limiting behaviors *z* → 1 and *η*_*ϕ*_ → 0 as *v* → 0. We conjecture the following explanation. As noted in Section “Theoretical analysis near perfect nesting”, we assume that the renormalized value of *v* is close to the bare one. However, as we decrease *v* we are not tuning *g*, so the bare value of *λ* = *g*^2^*c*^2^/*v* is increasing, not staying fixed. This increases the RG-time needed to flow to the one-dimensional manifold discussed in Section “Theoretical analysis near perfect nesting”. The large bare value of the effective coupling *λ* might be renormalizing *v* to larger values, before the one-dimensional manifold it reached. Remedying this requires decreasing *g* with decreasing *v* such that *λ* remains fixed. This task is beyond the scope of this paper, and we leave it for future work.

**Fig. 9:**
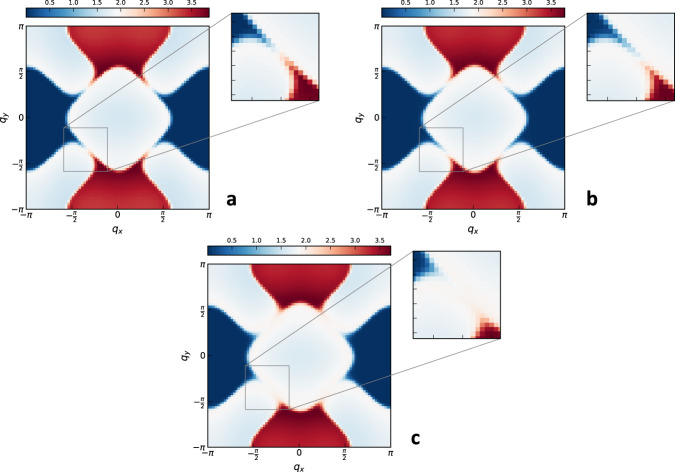
Measured occupation number. The total occupation number ${n}_{{{{{{{{\boldsymbol{q}}}}}}}}}={\sum }_{\alpha,\sigma }\langle {\psi }_{\alpha,\sigma,{{{{{{{\boldsymbol{q}}}}}}}}}^{{{{\dagger}}} }{\psi }_{\alpha,\sigma,{{{{{{{\boldsymbol{q}}}}}}}}}\rangle$ for the (**a**) free theory, and the interacting theory (**b**) at criticality (*r* = *r*_*c*_ ≈ 1.26) and (**c**) slightly in the ordered phase (*r* = 1.15). This explicitly shows the renormalization of the Fermi surface near the hot spots due to fluctuations of the order parameter. All plots are for *v* = *v*_1_ and *β* = 20, *L* = 80.

Finally, we show the fermion occupation function at criticality in Fig. 9 (as well as Supplementary Note 1), where we can see the renormalization of the hot-spots by the Yukawa interaction as well as the gap appearing at the hot-spots in the ordered phase. Unfortunately, within our spatial resolution it is not possible to measure the renormalized nesting parameter *v*, and therefore confirm the conjecture from the above paragraph.

## Discussion

In this work, we study the critical theory of the O(3) spin-fermion model as a model of the antiferromagnetic transition in two-dimensional metals. We use a novel HMC method that we extensively develop. Below we discuss some consequences of our work and outlook.

### Implications of theoretical results

Our study of the critical spin susceptibility *χ*(*ω*, ***q***) provides one of the few controlled numerical studies of a model where there is an observed violation of Hertz-Millis scaling. The critical exponents and C_4_-symmetric form of *χ*(*ω*, ***q***) can be directly tested experimentally with neutron scattering in materials where the bare hot-spot nesting value *v*_*B*_ is small.

Additionally, we provide strong evidence that the physics of the quantum critical metal is governed by the fixed point of ref. ^12^, even for appreciable nesting parameter values *v*. If one believes this evidence, the implication is that the theory of ref. ^12^, valid at small *v*, augmented with numerical calculations at larger *v*, can be used to generate a fully controlled quantitative solution to the problem. For example, non-equilibrium properties such as conductivity, which are generally very difficult to resolve with imaginary time methods, but are the most common observable used to experimentally identify the quantum critical fan, can be computed using small *v* perturbation theory about the fixed point of ref. ^12^ and supplemented with non-perturbative numerical calculation of the critical exponents. If valid, this approach would provide an unprecedented advance in the understanding of experimentally observed strange metals proximate to itinerant antiferromagnetism, characterized by, e.g., linear-in-temperature DC resistivity and present in many materials displaying unconventional superconductivity.

### Future directions with HMC

As noted in the main text, Fig. 6 does not convincingly show that *z* → 1 as *v* → 0, and the most likely reason for this is the increasing value of *λ* = *g*^2^*c*^2^/*v* as we decrease *v*. It is therefore desirable to revisit this problem, while keeping *λ* fixed by tuning *g*. We intend to address this in a future work.

In this work, we focus on only two critical exponents of the spin-fermion model, *z* and *η*_*ϕ*_, for the most straightforward comparison to the nearly-nested fixed point theory of ref. ^12^. There are several other pieces of critical data that remain to be examined for a more comprehensive comparison. The two most important ones are the critical exponent ${\nu}$ that relates the correlation length to the deviation from the critical point, and the scaling of the fermion Green’s function. Measuring the critical scaling of thermodynamic quantities like specific heat is also an important task. One can also tune *g* to a large enough value to see the onset of superconductivity in order to track the dependence of *T*_*c*_ on *v* and compare to the prediction of *T*_*c*_(*v*) made in ref. ^12^. Finally, here we only study the parameter values *u* = 0, *c* = 3, and five small values of *v*. An obvious question is how the conclusions of this work change with varying parameters *u*, *c*, as well as larger values of *v*.

The HMC algorithm we use in this work is quite general and can be applied to many other theories of interest. The main restrictions are that the configuration (boson) field be continuous and that the number of fermions can be doubled without qualitatively changing the physics of the model (this is not necessary if the fermion matrix is purely real). Some immediate candidates are other sign-problem-free ‘designer’ models of various flavors of quantum criticality, such as easy-plane *XY*^17^ and *Z*_2_^31^ antiferromagnetic transitions in metals, the Ising nematic transition in metals^26^, fermions coupled to emergent gauge fields^55–61^, Kondo lattice physics^62–64^, disorder-averaged criticality^65^, Gross–Neveu–Yukawa criticality^66^, and models of flat band physics^67–70^. Other classes of models are Hubbard-type models and electron-phonon models, which have been recently studied^35–47,49,71,72^ using various HMC and DQMC algorithms. We note that for theories with dynamical gauge fields of continuous gauge groups or non-linear-sigma models, HMC is less efficient due to the trouble it has in sampling the various topological sectors via molecular dynamics. In these cases, perhaps an HMC augmented with generative model sampling might make for a very efficient sampling^73^. This issue is not present in the present model, since the magnitude of the O(3) bosonic field is not restricted.

### Potential algorithmic improvements

The implementation could benefit from more sophisticated approaches to solving the linear system arising from each integration step within the HMC algorithm. Currently, the computational cost of our preconditioned CG approach enjoys ideal scaling in the limit of large *L*. However, the scaling with respect to *N*_*τ*_ for Δ*τ* fixed, i.e., the zero-temperature limit, is superlinear with respect to Δ*τ*. Several approaches to similar linear solves have been introduced in the LQCD and condensed matter literatures that could pay dividends here. These include Hasenbuch preconditioning^40,74^, multigrid preconditioners and solvers^75^, and non-iterative ideas based on Schur complements^43^. These approaches should also help for larger values of the Yukawa coupling, when the preconditioner used in this work would be less effective. Moreover, a mixed-precision implementation of CG on the GPU could provide significant speedup^76^.

There is also room for further investigation into the HMC hyperparameter auto-tuning procedure, as there exist alternative criteria for tuning *ε* and *n*_leap_. For example, one could instead tune *ε* based on the average acceptance rate or based on the stability of the numerical integrator. Meanwhile, the tuning of *n*_leap_ is by far the most expensive part of the warmup phase. The current state-of-the-art approach in the statistics literature for tuning *n*_leap_ is called the No U-Turn Sampler (NUTS)^77^. Implementing NUTS in our setting might offer a similar advantage.

## Methods

### QMC path integral with fermions

In order to simulate the action of Eq. (2) we put the system on a finite square lattice of size *L* with periodic boundary conditions. The lattice spacing is set to one. The imaginary time direction is also discretized into *N*_*τ*_ slices, with the imaginary time spacing, or Trotter step, labeled as Δ*τ*. We set Δ*τ* = 0.1. Fermions and boson obey anti-periodicity and periodicity, respectively, in the time direction. As in any path integral quantum Monte Carlo method involving fermions, we start by writing out the partition function and performing the Grassmann integral over the fermionic fields:

16 $$Z=	 \int\,d{{{{{{{\boldsymbol{\phi }}}}}}}}\ {e}^{-{S}_{B}({{{{{{{\boldsymbol{\phi }}}}}}}})}\int\,d{\psi }^{*}d\psi \ {e}^{-{S}_{F}(\psi,{\psi }^{*})-{S}_{FB}({{{{{{{\boldsymbol{\phi }}}}}}}},\psi,{\psi }^{*})}\\=	 \int\,d{{{{{{{\boldsymbol{\phi }}}}}}}}\ {e}^{-{S}_{B}({{{{{{{\boldsymbol{\phi }}}}}}}})}\det {(D({{{{{{{\boldsymbol{\phi }}}}}}}}))}^{{N}_{f}}.$$

Here, *D*(***ϕ***) is the fermion matrix of size 2 ⋅ 2 ⋅ *N*_*τ*_ ⋅ *L* ⋅ *L* = 4*N*_*τ*_*L*^2^, where the two factors of 2 correspond to the band and spin indices. Since the fermionic action is diagonal in the flavor index *j*, *N*_*f*_ enters trivially as a power of the determinant. Component-wise *D*(***ϕ***) is given by

17 $$D{({{{{{{{\boldsymbol{\phi }}}}}}}})}_{(\alpha,s,\tau,x,y),({\alpha }^{{\prime} },{s}^{{\prime} },{\tau }^{{\prime} },{x}^{{\prime} },{y}^{{\prime} })}=	 {\delta }_{\alpha,{\alpha }^{{\prime} }}{\delta }_{s,{s}^{{\prime} }}\left\{{\delta }_{x,{x}^{{\prime} }}{\delta }_{y,{y}^{{\prime} }}\left({\delta }_{\tau,{\tau }^{{\prime} }}[-1-{{\Delta }}\tau \,{\mu }_{\alpha }]\right.\right.\\ 	 \left.\left.+{\delta }_{\tau+1,{\tau }^{{\prime} }}[1-2\,{\delta }_{\tau,{N}_{\tau }-1}]\right)-{\delta }_{\tau,{\tau }^{{\prime} }}\,{{\Delta }}\tau \,{t}_{\alpha,(x,y),({x}^{{\prime} },{y}^{{\prime} })}\right\}\\ 	+{\delta }_{x,{x}^{{\prime} }}{\delta }_{y,{y}^{{\prime} }}{\delta }_{\tau,{\tau }^{{\prime} }}\ {{\Delta }}\tau \ g\ {e}^{i{{{{{{{{\boldsymbol{Q}}}}}}}}}_{AF}\cdot (x,y)}\ {{{{{{{\boldsymbol{\phi }}}}}}}}(\tau,\,x,\,y)\cdot {{{{{{{{\boldsymbol{\tau }}}}}}}}}_{\sigma,{\sigma }^{{\prime} }}\,{\sigma }_{\alpha,{\alpha }^{{\prime} }}^{(x)},$$

which is read directly from the action in Eq. (2). Here, *τ* is in the range {0, …, *N*_*τ*_ − 1}, and *x*, *y* are in the range {0, …, *L* − 1}. *δ*_*a*,*b*_ is the Kronecker delta function. ${\sigma }_{\alpha,{\alpha }^{{\prime} }}^{(x)}$ is the usual Pauli matrix, but acting on the band indices instead of the spin indices.

### Stochastic formulation of the determinant

In the rest of this section, we will use the notation *ϕ* (not bold) to denote the bosonic field, to emphasize its interpretation alternatively as either a vector of length 3*N*_*τ*_*L*^2^ or an array of size 3 × *N*_*τ*_ × *L* × *L*, depending on context, as opposed to its equivalent representation as 3-component field ***ϕ*** = ***ϕ***(*τ*, *x*, *y*).

In the usual BSS algorithm^78^, the determinant in Eq. (16) is rewritten as

18 $$\det (D(\phi ))=\det \left({{{{{{{\bf{1}}}}}}}}+\mathop{\prod }\limits_{l=0}^{{N}_{\tau }-1}{B}_{l}(\phi )\right),$$

where **1** and the *B*_*l*_(*ϕ*) are square matrices of size 2 ⋅ 2 ⋅ *L* ⋅ *L* = 4*L*^2^. This reduces the cost of exact computation of the determinant from the naive scaling of ${{{{{{{\mathcal{O}}}}}}}}({(4{N}_{\tau }{L}^{2})}^{3})$ to the improved scaling of ${{{{{{{\mathcal{O}}}}}}}}({N}_{\tau }{(4{L}^{2})}^{3})$ for every configuration *ϕ*. Although this is better than the direct computation of the larger determinant, the scaling with respect to *L* is nonetheless quite severe.

As an alternative to the exact computation of the determinant, we use the pseudofermion method^79,80^ to evaluate it stochastically. This method is based on the expression

19 $$\det (A)\propto \int\,{{{{{{{\mathcal{D}}}}}}}}\varphi {{{{{{{\mathcal{D}}}}}}}}{\varphi }^{*}\ {e}^{-{\varphi }^{*}{A}^{-1}\varphi },$$

where *φ* is an auxiliary complex bosonic field (called a ‘pseudofermion’) and *A* is any Hermitian positive definite matrix.

Although the anti-unitary symmetry of ref. ^16^ guarantees that $\det (D(\phi ))$ is always non-negative, *D*(*ϕ*) itself is not necessarily positive definite. However, we can write

20 $$\det {(D)}^{{N}_{f}}=	 {(\det (D)\det {(D)}^{*})}^{{N}_{f}/2}\\=	 {(\det (D)\det ({D}^{{{{\dagger}}} }))}^{{N}_{f}/2}=\det {(D{D}^{{{{\dagger}}} })}^{{N}_{f}/2}.$$

The matrix *D*(*ϕ*)*D*(*ϕ*)^†^ is guaranteed to be positive definite. Note that when *N*_*f*_ is odd, the integral in Eq. (19) comes with a fractional power, which limits us to the case of even *N*_*f*_. From here on, we set *N*_*f*_ = 2, and moreover this value is used in our simulations. Rewriting the determinant in this way, the partition function becomes

21 $$Z=\int\,d\phi \,d{\varphi }^{*}d\varphi \ {e}^{-\left({{{{{{{{\mathcal{S}}}}}}}}}_{B}(\phi )+{{{{{{{{\mathcal{S}}}}}}}}}_{PF}(\phi,\varphi )\right)},$$

where ${{{{{{{{\mathcal{S}}}}}}}}}_{PF}(\phi,\,\varphi )={\varphi }^{*}{(D(\phi )D{(\phi )}^{{{{\dagger}}} })}^{-1}\varphi$. Note that the dimension of *φ* is the same as *D*(*ϕ*), i.e., 4*N*_*τ*_*L*^2^. This new partition function defines a joint distribution *p*(*ϕ*, *φ*), which can be sampled with Markov chain Monte Carlo (MCMC).

### Solving the linear system

While Eq. (21) introduces a matrix inverse to the action, the expression can be evaluated efficiently by treating the application of ${(D{D}^{{{{\dagger}}} })}^{-1}$ to *φ* as the solution to a linear system:

22 $$(D{D}^{{{{\dagger}}} })\eta=\varphi,$$

where *η* is the unknown. A wide class of iterative solvers are available to tackle this problem efficiently. Additionally, other non-iterative solvers have recently been employed in the application of HMC to the Hubbard model^43^. Here, we use the conjugate gradient (CG) method^81^, a commonly used technique with practical advantages for Hermitian positive definite systems.

#### Choice of preconditioner

Iterative solvers can often be preconditioned with a transformation that improves the conditioning of the linear system. For any preconditioner *P*, the equation *P*^−1^(*DD*^†^)*η* = *P*^−1^*ϕ* has the same solution as the original system but may be better conditioned. Within the CG algorithm, we choose a preconditioner as follows.

Note that *D*(*ϕ*) as defined in Eq. (17) can be split into two terms,

23 $$D(\phi )=K+V(\phi ),$$

where the first term, *K*, i.e. the fermionic kinetic energy, consists of all terms in Eq. (17) that are independent of *ϕ*. Since *K* is a translation-invariant operator (modulo fermionic imaginary-time antiperiodicity), it can be diagonalized and inverted efficiently using fast Fourier operations and then used to construct an efficient preconditioner.

To wit, let ${{{{{{{\mathcal{F}}}}}}}}$ denote the discrete Fourier transform with respect to the spacetime lattice indices (*τ*, *x*, *y*), and let ${{{{{{{\mathcal{T}}}}}}}}$ be the unitary diagonal ‘twist’ operator defined by

24 $${{{{{{{{\mathcal{T}}}}}}}}}_{(\alpha,s,\tau,x,y),({\alpha }^{{\prime} },{s}^{{\prime} },{\tau }^{{\prime} },{x}^{{\prime} },{y}^{{\prime} })}={\delta }_{(\alpha,s,\tau,x,y),({\alpha }^{{\prime} },{s}^{{\prime} },{\tau }^{{\prime} },{x}^{{\prime} },{y}^{{\prime} })}\,{e}^{\pi i\tau }.$$

Note that ${{{{{{{\mathcal{T}}}}}}}}$ transforms imaginary-time-periodic fields to imaginary-time-antiperiodic fields, and as such we can write

25 $$K=\widetilde{{{{{{{{\mathcal{F}}}}}}}}}\,\widehat{K}\,{\widetilde{{{{{{{{\mathcal{F}}}}}}}}}}^{*},$$

where $\widetilde{{{{{{{{\mathcal{F}}}}}}}}}:={{{{{{{\mathcal{T}}}}}}}}{{{{{{{\mathcal{F}}}}}}}}$ is the ‘twisted’ Fourier transform and $\widehat{K}$ is diagonal.

Our preconditioner is then defined by

26 $$P:={(K{K}^{{{{\dagger}}} })}^{-1}=\widetilde{{{{{{{{\mathcal{F}}}}}}}}}\,\widehat{P}\,{\widetilde{{{{{{{{\mathcal{F}}}}}}}}}}^{*},$$

where $\widehat{P}=\widehat{K}{\widehat{K}}^{{{{\dagger}}} }$ is diagonal. As such, *P* can be applied with computational cost that is linear (up to log factors) in the spacetime volume *N*_*τ*_*L*^2^. In the limit where the coupling constant *g* → 0, this is the perfect preconditioner, $P={(D{D}^{{{{\dagger}}} })}^{-1}$, achieving convergence in a single CG iteration. More generally, the preconditioner is effective in addressing poor conditioning due to the unboundedness of the differential operator term of *D* as the imaginary time step Δ*τ* is refined, in analogy with, e.g., Laplacian preconditioners^82^ in the numerical PDE literature.

See also ref. ^35^ for a similar approach to preconditioning based on a noninteracting model. Note that such a preconditioner may not be satisfactory in general in the strong coupling limit, and we discuss possible future improvements in Section “Potential algorithmic improvements”. For the couplings studied in this paper, we do nonetheless observe improvement due to the preconditioner, as detailed in Supplementary Note 5.

### HMC

Hybrid/Hamiltonian Monte Carlo (HMC) is a Markov chain Monte Carlo (MCMC) method in which numerical integration of Hamilton’s equations in a fictitious phase space produces sequential updates in a Markov chain^50,83^. Historically used as tool for the study of lattice quantum chromodynamics (LQCD), HMC is currently the state-of-the-art method in the field, where it has yielded sub-1% errors on over ~10^10^ degrees of freedom^75,84^.

We now give a brief overview of HMC as applied to theories with fermions. As explained above, Eq. (21) defines a joint probability distribution,

27 $$p(\phi,\,\varphi )=\frac{1}{Z}{e}^{-{{{{{{{{\mathcal{S}}}}}}}}}_{B}(\phi )-{{{{{{{{\mathcal{S}}}}}}}}}_{PF}(\phi,\varphi )},$$

that needs to be sampled to estimate observables. We adopt a Gibbs sampler approach to sampling the two fields *ϕ*, *φ*, wherein we update each one conditioned on the other in alternating fashion.

The *φ* sample can be drawn exactly from the conditional distribution for fixed *ϕ*, which is the complex normal distribution ${{{{{{{\mathcal{N}}}}}}}}(0,\,D(\phi )D{(\phi )}^{{{{\dagger}}} })$. This is achieved by sampling a standard complex Gaussian vector *χ* and setting *φ* = *D*(*ϕ*)*χ*.

Because the action is not quadratic in *ϕ*, sampling of this field remains non-trivial but can be handled with MCMC. We employ the HMC method for this purpose. The first step is to introduce a field *π* that acts as an artificial conjugate momentum to the field *ϕ*. Together, *ϕ* and *π* define phase space coordinates for a fictitious Hamiltonian system

28 $$\tilde{H}(\phi,\,\varphi,\,\pi )=K(\pi ;M)+{{{{{{{\mathcal{S}}}}}}}}(\phi,\,\varphi )=\frac{1}{2}{\pi }^{\top }{M}^{-1}\pi+{{{{{{{\mathcal{S}}}}}}}}(\phi,\,\varphi ),$$

consisting of ‘kinetic energy’ and ‘potential energy’ terms. The kinetic energy specifically is defined with respect to a choice of metric *M*, also called a mass matrix, which is given to be positive definite. The choice of *M* will be discussed later. This Hamiltonian allows us to define a new joint distribution $p(\phi,\,\varphi,\,\pi ) \sim {e}^{-\tilde{H}(\phi,\varphi,\pi )}$, compatible with our target distribution after marginalization of *π*.

The new joint distribution can now be efficiently sampled by drawing *π* directly as a normal random vector with covariance *M* and then integrating the equations of motion of $\tilde{H}$,

29 $$\frac{d\phi }{dt}={M}^{-1}\pi \quad {{{{{{{\rm{and}}}}}}}}\quad \frac{d\pi }{dt}=-\frac{\partial {{{{{{{\mathcal{S}}}}}}}}(\phi,\,\varphi )}{\partial \phi }.$$

Here, *t* is a fictitious ‘time’ variable. The trajectory defined by Eqs. (29) is also known as the molecular dynamics trajectory. Once a new sample of (*ϕ*, *π*) is obtained, its *ϕ* component is taken as the new sample of *p*(*ϕ*, *φ*).

With these tools, we can construct the Markov chain update for *ϕ* as follows:

1. Generate a momentum sample from $\pi \sim {{{{{{{\mathcal{N}}}}}}}}(0,\,M)$.
2. Approximately integrate the molecular dynamics (29) with initial condition (*ϕ*, *π*) for some time to produce new configuration $({\phi }^{{\prime} },\,{\pi }^{{\prime} })$.
3. Accept the new configuration (${\phi }^{{\prime} },\,{\pi }^{{\prime} }$) with probability $\alpha=\min \left(1,\,{e}^{-\left\{\tilde{H}({\phi }^{{\prime} },{\pi }^{{\prime} })-\tilde{H}(\phi,\pi )\right\}}\right)$.

Exact integration of Eqs. (29) is not feasible and must therefore be performed numerically in discrete time. A suitable choice of numerical integration scheme is leapfrog integration^85^, which preserves phase space volume and ensures that the final Metropolis accept/reject step preserves detailed balance.

### Scale-invariant sampling

In high dimensions, local MCMC methods encounter difficulties in the setting of ‘poorly scaled,’ i.e., severely anisotropic, distributions. In order to guarantee a nonvanishing acceptance rate without correcting for this difficulty, the local moves in the MCMC sampler are constrained by the smallest scale present in the problem, and as such the autocorrelation time with respect to the large scales must grow accordingly. For a critical lattice model, the smallest scale present in the distribution is not bounded away from zero in the thermodynamic limit, and this difficulty must be addressed to maintain constant autocorrelation time in this limit. Existing approaches based on affine invariance are unsuitable in this context due either to a curse of dimensionality^86^ or to unacceptable (i.e., at least quadratic) scaling^87^ with respect to the spacetime volume *N*_*τ*_*L*^2^. Relative to these approaches, we are able to exploit the known a priori structure of our model, i.e., translation invariance, to achieve a fast scale-invariant sampler. Specifically, within the context of HMC, we learn an optimal metric *M* ‘online.’ In contrast with^44^, beyond translation invariance we do not assume any a priori functional form for the metric, as any a priori choice may struggle near criticality. Since *M* is updated adaptively, it is then important to tune the HMC hyperparameters *ε* and *n*_nleap_ adaptively in a scale-invariant fashion. For this task we adapt best practices from the statistics literature (as implemented, e.g., in ref. ^52^) to our setting, as we shall discuss below. These quantities (i.e., *M*, *ε*, and *n*_leap_) are all determined during a warm-up phase, after which they are fixed for the duration of the run.

#### Online metric estimation

HMC as described above allows for an arbitrary choice of metric or mass matrix *M*. When the underlying probability distribution to be sampled is poorly scaled, the metric must be adapted to the scaling of the distribution to maintain a constant autocorrelation time. An idealized choice for *M* is given by the inverse of the exact covariance matrix Σ_true_: = 〈*ϕϕ*^⊤^〉 of the underlying distribution *p*, i.e., by $M={{{\Sigma }}}_{{{{{{{{\rm{true}}}}}}}}}^{-1}$. In the case where *p* is the Gaussian ${{{{{{{\mathcal{N}}}}}}}}(0,\,{{{\Sigma }}}_{{{{{{{{\rm{true}}}}}}}}})$, such a choice is equivalent to rescaling *p* to the standard (isotropic) Gaussian distribution ${{{{{{{\mathcal{N}}}}}}}}(0,\,{{{{{{{\bf{1}}}}}}}})$. More generally, such a choice can be viewed as rescaling *p* to a distribution with unit covariance matrix.

There are two obstacles to achieving this idealized choice. First, the exact covariance Σ_true_ is not known a priori. Second, even if it were known or estimated as Σ ≈ Σ_true_, the cost of even storing this matrix scales as $\sim {(V{N}_{\tau })}^{2}$, which already imposes a computational bottleneck. Worse still, within the HMC algorithm, we must generate samples from ${{{{{{{\mathcal{N}}}}}}}}(0,\,{{{\Sigma }}}^{-1})$, which in general scales as $\sim {(V{N}_{\tau })}^{2}$, i.e., as the cost of a Cholesky factorization of Σ.

The first of these obstacles can be tackled via online estimation of the covariance matrix Σ_true_ from samples. To wit, we can choose some initialization for Σ (typically Σ = **1**) and run the HMC algorithm to produce a batch of *S* samples *ϕ*^(1)^, …, *ϕ*^(*S*)^ of the bosonic field, then reset ${{\Sigma }}\leftarrow {{{\Sigma }}}_{{{{{{{{\rm{est}}}}}}}}}:=\frac{1}{S}\mathop{\sum }\nolimits_{s=1}^{S}{\phi }^{(s)}{\phi }^{(s),T}$ and repeat the procedure until Σ converges.

There is an appearance of circular reasoning to such an online estimation scheme in that it assumes the ability to draw samples *ϕ* ~ *p* via our MCMC sampler (which may be bottlenecked by a long autocorrelation time) in order to improve the autocorrelation of the MCMC method itself! However, it is important to realize that in the initial iteration, the autocorrelation time for the smallest eigenmodes of Σ_true_ is not long. Indeed, to get a nonvanishing acceptance probability, the step size in HMC must be tuned to be on the order of the smallest eigenvalue of the covariance. Due to anisotropy, this may result in very slow MCMC mixing in the highest eigendirections, but this does not interfere with our ability to estimate the smallest eigenmodes. After the first iteration, having corrected for the smallest eigenmodes, the procedure proceeds to correct the next smallest, etc.

Despite its theoretical appeal, there are two problems with this approach, from the point of view of computational scaling. First, to get an estimate Σ_est_ ≈ Σ_true_ of fixed accuracy requires a size-extensive number *S* of samples, due to the size-extensive numerical rank of Σ_true_. Second, as mentioned earlier, the cost of storing and factorizing Σ_est_ is prohibitive. In fact, both problems can be addressed by exploiting translation invariance.

Indeed, since Σ_true_ is translation-invariant, it is equivalently diagonal in Fourier space—or more precisely, (3 × 3)-block-diagonal with respect to the vector index *l* = 1, 2, 3 of ***ϕ***. Therefore ${{{\Sigma }}}_{{{{{{{{\rm{true}}}}}}}}}={{{{{{{\mathcal{F}}}}}}}}\,{\widehat{{{\Sigma }}}}_{{{{{{{{\rm{true}}}}}}}}}\,{{{{{{{{\mathcal{F}}}}}}}}}^{*}$, where ${{{{{{{\mathcal{F}}}}}}}}$ represents the discrete Fourier transform with respect to the spacetime lattice indices and ${\widehat{{{\Sigma }}}}_{{{{{{{{\rm{true}}}}}}}}}$ is block-diagonal. As such the block-diagonal can be obtained as the expectation ${{{{{{{\rm{diag}}}}}}}}({[{\widehat{{{\Sigma }}}}_{{{{{{{{\rm{true}}}}}}}}}]}_{l{l}^{{\prime} }})=\langle {\widehat{\phi }}_{l}\odot \overline{{\widehat{\phi }}_{{l}^{{\prime} }}}\rangle$, where samples $\widehat{\phi }$ are obtained as $\widehat{\phi }={{{{{{{\mathcal{F}}}}}}}}\phi$ from samples *ϕ* and ‘⊙’ indicates the entrywise product. In practice, for simplicity we consider only the true diagonal of $\widehat{{{\Sigma }}}$, which is sufficient to address the increasing anisotropy due to large volume. Concretely, we therefore form

30 $$\widehat{\sigma }\leftarrow {\widehat{\sigma }}_{{{{{{{{\rm{est}}}}}}}}}:=\frac{1}{S}\mathop{\sum }\limits_{s=1}^{S}\widehat{\phi }\odot \overline{\widehat{\phi }}$$

and let ${{\Sigma }}={{{{{{{\mathcal{F}}}}}}}}\,\widehat{{{\Sigma }}}\,{{{{{{{{\mathcal{F}}}}}}}}}^{*}$, where $\widehat{{{\Sigma }}}$ is the diagonal matrix with diagonal $\widehat{\sigma }$.

In order to draw sample momenta $\pi \sim {{{{{{{\mathcal{N}}}}}}}}(0,M)$ within the HMC algorithm, observe that $M={{{\Sigma }}}^{-1}=({{{{{{{\mathcal{F}}}}}}}}\,{\widehat{{{\Sigma }}}}^{-1/2}){({{{{{{{\mathcal{F}}}}}}}}{\widehat{{{\Sigma }}}}^{-1/2})}^{*}$. Therefore we can simply draw $z \sim {{{{{{{\mathcal{N}}}}}}}}(0,\,{{{{{{{\bf{1}}}}}}}})$ and let $\pi={{{{{{{\mathcal{F}}}}}}}}({\widehat{\sigma }}^{-1/2}\odot z)$, where ${\widehat{\sigma }}^{-1/2}$ indicates the entrywise inverse square root of $\widehat{\sigma }$. The total cost of drawing such a sample is linear in the spacetime volume *L*^2^*N*_*τ*_, up to a log factor, as is the cost of evaluating the HMC energy of Eq. (28).

Since the off-diagonal elements of $\widehat{{{\Sigma }}}$ are known a priori to be zero (and implicitly set to zero automatically), we only need *O*(1) effective samples to estimate $\widehat{\sigma }$ to fixed accuracy. Hence the total cost of each iterative update of $\widehat{{{\Sigma }}}$ scales linearly in the spacetime volume *L*^2^*N*_*τ*_, up to log factors.

#### Online HMC hyperparameter tuning

Within the HMC algorithm, once the metric *M* is fixed, it remains to choose (subordinate to this choice) appropriate values for the integration step size *ε* > 0 and the number *n*_leap_ of leapfrog integration steps per proposal.

First we consider the choice of *ε*. Roughly speaking, one wants to choose *ε* as large as possible without sabotaging the acceptance rate. Quantitatively, let *α*(*ε*) denote the expected acceptance probability for a single leapfrog integration step of size *ε*. More precisely, recalling that $\alpha=\alpha (\phi,\,\pi,\,{\phi }^{{\prime} },\,{\pi }^{{\prime} })$ denotes the acceptance probability of a proposed move $(\phi,\,\pi )\to ({\phi }^{{\prime} },\,{\pi }^{{\prime} })$, we may define $\alpha (\varepsilon )={\langle \alpha (\phi,\,{\phi }^{{\prime} })\rangle }_{\varepsilon,{n}_{{{{{{{{\rm{leap}}}}}}}}}=1}$, where the statistical expectation is computed by sampling (*ϕ*, *π*) ~ *p* and then sampling $({\phi }^{{\prime} },\,{\pi }^{{\prime} })$ as a proposal from *ϕ* according to the HMC algorithm with step size *ε* and *n*_leap_ = 1. Then one wants to maximize *ε* subject to the inequality

31 $${(1-\alpha (\varepsilon /2))}^{2}\le 2(1-\alpha (\varepsilon )).$$

The left-hand side estimates the probability of accepting two steps of size *ε*/2, whereas the right-hand side estimates the probability of accepting one step of size *ε*. Since the former strategy costs twice as many linear solves ${(D{D}^{{{{\dagger}}} })}^{-1}\varphi$ as the latter, the latter is computationally preferable as long as the inequality holds.

In practice, we maintain an estimate for *α*(*ε*) and *α*(*ε*/2) based on an empirical average over a recent history of samples, and we increase or decrease *ε* when Eq. (31) is satisfied or violated, respectively.

Now we turn to the choice of *n*_leap_. Within the actual HMC algorithm, we choose *n*_leap_ uniformly at random from the set $\{1,\ldots,{n}_{\max }\}$, independently for each iteration, where ${n}_{\max }$ is a hyperparameter to be determined adaptively. This ‘jittering’ procedure is standard in the statistics community^52^. The hyperparameter ${n}_{\max }$ is determined as the maximizer of the expected squared jump distance (ESJD)^51^

32 $${{{{{{{\bf{ESJD}}}}}}}}(n):={\left\langle {(\phi -{\phi }^{{\prime} })}^{\top }M(\phi -{\phi }^{{\prime} })\,\alpha (\phi,\,{\phi }^{{\prime} })\right\rangle }_{\varepsilon,{n}_{{{{{{{{\rm{leap}}}}}}}}}=n}.$$

Importantly, the metric *M* is needed here to correctly define the squared distance ${\left|\phi -{\phi }^{{\prime} }\right|}_{M}^{2}={(\phi -{\phi }^{{\prime} })}^{\top }M\,(\phi -{\phi }^{{\prime} })$.

In practice, similarly to *α*(*ε*), we maintain an estimate for **ESJD**(*n*) based on an empirical average over recent history, and we choose ${n}_{\max }$ to maximize it.

During the warmup phase, the three components of $\varepsilon,\,{n}_{\max }$ and *M* are each tuned several times, until they stop changing appreciably.

Note that since the acceptance probability in HMC is directly determined by the energy conservation error in the integration of Hamilton’s equations (29), alternative higher-order integration schemes besides leapfrog can be considered to improve acceptance rates at the price of more expensive integration steps, cf. for example^88,89^.

### Estimation of observables

Here we describe the methods we use to compute physical observables directly in Fourier space, while keeping the computational cost near-linear in *βV*.

#### Bosonic observables

Bosonic observables can be computed directly as a sample average over the bosonic field variable *ϕ*. Specifically of interest is the SDW susceptibility

33 $$\chi (\omega,\,{{{{{{{\boldsymbol{q}}}}}}}})=\int\nolimits_{0}^{\beta }\mathop{\sum}\limits_{{{{{{{{\boldsymbol{r}}}}}}}}}\langle {{{{{{{\boldsymbol{\phi }}}}}}}}(\tau,\,{{{{{{{\boldsymbol{r}}}}}}}})\cdot {{{{{{{\boldsymbol{\phi }}}}}}}}(0,\,{{{{{{{\boldsymbol{0}}}}}}}})\rangle {e}^{i\omega \tau -i{{{{{{{\boldsymbol{q}}}}}}}}\cdot {{{{{{{\boldsymbol{r}}}}}}}}}\,d\tau .$$

The SDW susceptibility can be estimated at linear cost (up to log factors) as

34 $$\chi (\omega,\,{{{{{{{\boldsymbol{q}}}}}}}})=\left\langle|\widehat{\phi }(\omega,\,{{{{{{{\boldsymbol{q}}}}}}}}){|}^{2}\right\rangle \,{{\Delta }}\tau,$$

where we recall that $\widehat{\phi }={{{{{{{\mathcal{F}}}}}}}}\phi$ is the spacetime discrete Fourier transform of *ϕ*.

#### Fermionic observables

Fermionic observables require more care to estimate while maintaining almost-linear scaling. Of particular interest are the two-point correlator (in Fourier space)

35 $${G}_{\alpha,s}(\omega,\,{{{{{{{\boldsymbol{q}}}}}}}})=\int\nolimits_{0}^{\beta }\mathop{\sum}\limits_{{{{{{{{\boldsymbol{r}}}}}}}}}\langle {\psi }_{\alpha,s}(0,\,{{{{{{{\boldsymbol{0}}}}}}}}){\psi }_{\alpha,s}{(\tau,\,{{{{{{{\boldsymbol{r}}}}}}}})}^{*}\rangle {e}^{i\omega \tau -i{{{{{{{\boldsymbol{q}}}}}}}}\cdot {{{{{{{\boldsymbol{r}}}}}}}}}\,d\tau .$$

and the superfluid (SF) density, which is defined in Supplementary Note 3.

The key observation is that all fermionic observables of interest can be phrased in terms of the expectation value of the diagonal of a matrix for which it is possible to perform efficient matrix-vector multiplications, i.e., as

36 $$\langle {{{{{{{\rm{diag}}}}}}}}({O}_{\phi })\rangle,$$

where the statistical average is taken with respect to the bosonic density for *ϕ* and where *O*_*ϕ*_ is an operator that depends on the bosonic field. Rather than compute the diagonal entries individually, it is more efficient to recover them simultaneously via the identity

37 $${{{{{{{\rm{diag}}}}}}}}({O}_{\phi })={\mathbb{E}}[v\odot ({O}_{\phi }v)],$$

where *v* is a random vector with independent entries that take values ±1 each with probability 1/2 and ‘${\mathbb{E}}$’ indicates the expectation with respect to this distribution over *v*. As before, ‘⊙’ indicates the entrywise product of vectors. This identity defines a randomized matrix-free algorithm for recovering a matrix diagonal from only *O*(1) matrix-vector multiplications, which has appeared before in various works^90,91^. Moreover, after taking traces of both sides, one recovers the famous Hutchinson trace estimator^92^. During the preparation of this work, this randomized diagonal estimator has also appeared in ref. ^44^ for the same purpose of computing fermionic observables, though their approach to computing quartic fermionic observables such as the SF density differ somewhat from ours.

Since we must average diag(*O*_*ϕ*_) over the bosonic distribution for *ϕ*, we can in fact obtain a consistent estimator by independently drawing a single vector *v*^(*s*)^ for each bosonic sample *ϕ*^(*s*)^, *s* = 1, …, *S*, where *S* is the sample size of our empirical average and estimating

38 $${{{{{{{\rm{diag}}}}}}}}({O}_{\phi })\,\approx \,\frac{1}{S}\mathop{\sum }\limits_{s=1}^{S}{v}^{(s)}\odot {O}_{\phi }{v}^{(s)}.$$

Further details of the implementation of the approach to computing fermionic observables (i.e., the specification of *O*_*ϕ*_ for observables of interest) can be found in Supplementary Note 3.

### Numerical performance

Here we summarize the numerical performance of our algorithm, with more details provided in Supplementary Note 6. As noted in the Introduction, in the presence of a critical slowing down, the HMC algorithm can require a number of integration steps per effective sample of $O({\beta }^{1/4+{z}_{1}}{V}^{1/4+{z}_{2}})$, where *z*_1_, *z*_2_ are to be determined empirically. To extract these exponents, we benchmark our algorithm across spacetime lattice sizes at the critical parameters of the theory, scaling with respect to *V* = *L*^2^ and *N*_*τ*_ separately. We track the growth of the integrated autocorrelation time *τ*_int_ of the total SDW susceptibility *χ* ≡ *χ*(0, 0) at criticality, and use it to quantify the wallclock time and number of HMC integration steps per effective sample: *τ*_int_ × *n*_leap_.

We find that our algorithm exhibits constant scaling of *τ*_int_ with respect to both lattice volume and inverse temperature. For the exponents, we find *z*_1_ ≈ 0.5, *z*_2_ ≈ 0. This implies an absence of critical slowing down with respect to the lattice volume *V* for the auto-tuned HMC algorithm presented in this work.

The actual wall clock time in turn depends on the cost of the linear solves of the form Eq. (22). With the preconditioned CG approach described in Section “Choice of preconditioner”, we observe near linear scaling in wall clock time per effective sample with respect to *V*, while the scaling is superlinear with respect to *N*_*τ*_. The linear solve can be approached with more advanced techniques, as well as more refined GPU parallelism. These directions are discussed in Section “Potential algorithmic improvements”.

## Supplementary information

Supplementary Information

Peer Review File

## Data Availability

The data analyzed in the current manuscript is available from the corresponding author upon reasonable request.

## Code availability

All the custom codes used in this study can be requested from the corresponding author.
