# Supplementary material for: Non-Hertz-Millis scaling of the antiferromagnetic quantum critical metal via scalable Hybrid Monte Carlo
Source: Nat Commun. 2023 May 3;14:2547. doi: 10.1038/s41467-023-37686-4 (PMC10156689; doi:10.1038/s41467-023-37686-4)
Supplement: Supplementary file 1 — Supplementary Information [file 41467_2023_37686_MOESM1_ESM.pdf]

Supplementary Information for “Non-Hertz-Millis scaling of the antiferromagnetic quantum critical metal via scalable Hybrid Monte Carlo”

Peter Lunts,* Michael S. Albergo, and Michael Lindsey

SUPPLEMENTARY NOTE 1. DETAILS OF RESULTS

Here we provide more details and supplementary plots for our data analysis, the results of which are presented in the Results Section.

A. Phase diagram

The phase diagram of Fig. 4 is obtained by first identifying the location of the thermal ‘phase transition,’ $r_c(T)$, at various temperatures. Due to the Mermin-Wagner theorem, this transition is actually a crossover. However, a sharp $r_c(T)$ can still be extracted from the finite-size behavior of the Binder cumulant. The Binder cumulant of the order parameter $\boldsymbol{\phi}$ is defined as $B_c \equiv 1 - \frac{3\langle \mathbf{\Phi}^4 \rangle}{5\langle \mathbf{\Phi}^2 \rangle^2}$, where $\mathbf{\Phi} \equiv \sum_{\tau,x,y} \boldsymbol{\phi}(\tau, x, y)$. We show an example plot in Fig. 1 for $v = v_1$ and $\beta = 10$. The transition point $r_c(T)$ is defined as the value of r at which all the different L curves cross (at least for large enough L). In Supplementary Note 4 we show that the ‘would-be’ transition is indeed second-order. Examining the curves in Supplementary Fig. 1 by eye gives us a value $r_c(T)$ with an error $\Delta r_c(T)$.

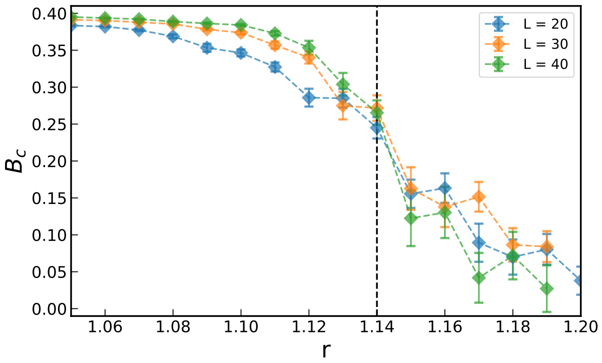

Supplementary Fig. 1: **Crossing of the Binder cumulant for different L.** Binder cumulant B_c as a function of r for $v = v_1$, $\beta = 10$. It goes from $B_c = 0$ in the disordered phase to $B_c = 0.4$ in the ordered phase. The ‘critical point’ is identified as the crossing point of the different L curves, here taken to be $r_c(T) = 1.14 \pm 0.01$.

Once $r_c(T)$ is obtained for several T values, we extrapolate them to $T = 0$ using a simple polynomial fit, $r_c(T) = r_c - a\,T^b$, which gives us our estimate of the quantum critical point $r_c \equiv r_c(T = 0)$. The values of r_c that we obtain for v_i, $i = 1, \ldots, 5$ are all given by $r_c \approx 1.2554$. Putting everything together gives the phase diagram in Fig. 4. The error bars denote the one sigma confidence interval.

To probe for superconductivity we use the method of Ref. [1]. We observe no superconductivity at r_c down to the lowest temperature that we study, $T = 1/80$. We give more details on this computation in Supplementary Note 2.

In Supplementary Fig. 2 we show plots of the occupation function for more nesting parameter values, namely $v = v_5$.

* plunts@umd.edu

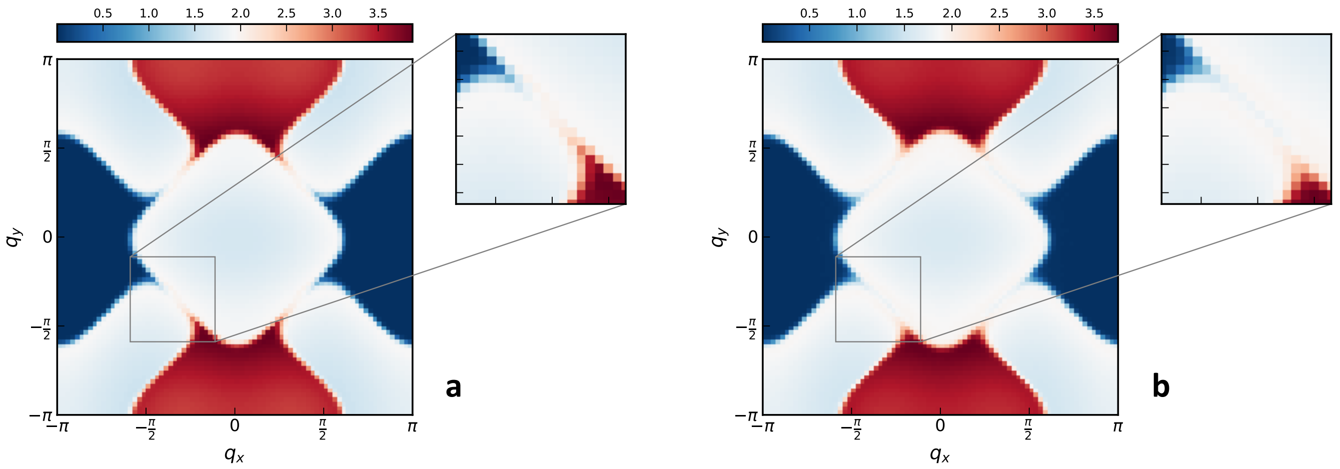

Supplementary Fig. 2: **Measured occupation number.** The total occupation number $n_{\boldsymbol{q}} = \sum_{\alpha,\sigma} \langle \psi^\dagger_{\alpha,\sigma,\boldsymbol{q}} \psi_{\alpha,\sigma,\boldsymbol{q}} \rangle$ for the (a) free theory and the (b) interacting theory at criticality ($r = r_c$). Both plots are at $v = v_5, \beta = 20, L = 80$.

B. Scaling of spin susceptibility at criticality

Having established the phase diagram and located the QCP, we examine the boson correlation function at criticality. We set $r = 1.26 \gtrsim r_c$, which is very slightly further into the disordered phase than the extrapolated value r_c. This is done to stay on the safe side of the transition, i.e. within the critical fan.

The log-log plots of $\chi^{-1}(\omega) - \chi^{-1}(0)$ for $\beta = 80, L = 20$ and of $\chi^{-1}(q_x) - \chi^{-1}(0)$ for $\beta = 20, L = 80$ are shown in Fig. 5a and Fig. 5b, respectively. As noted there, the regions of intermediate algebraic scaling are chosen by eye. For all nesting parameter values we define the regions as $10\pi/\beta \le \omega < 2.0$ and $8\pi/L \le q_x < 2.3$. The slope of the linear fits in these regions determine the critical exponents z and η_ϕ, shown in Fig. 6. To compute the error bars, we used a bootstrap method, where the selected data points were re-sampled 10000 times, allowing for duplicates.

The symmetry of the full spatial dependence is seen from Fig. 7 for $v = v_5$. In Supplementary Fig. 3 we provide similar zoomed-in plots for the other v_i, showing the C_4 symmetry at small momenta. In Supplementary Fig. 4, we also show that the contour at the same small momenta only get more 'square-like' with increasing L, indicating that the emergent C_4 symmetry is not a finite-size effect but a true long-wavelength feature of the theory in the thermodynamic limit.

SUPPLEMENTARY NOTE 2. SUPERFLUID DENSITY

In order to test for superconductivity in our system, we apply the method of Ref. [1] to compute the superfluid density ρ_s. The system is a superconductor if ρ_s surpasses the universal BKT value of $\Delta\rho_s = 2T/\pi$.

The current density operator in the $\hat{x}$ direction is given by

$$j^{(\hat{x})}_{\tau,x,y} = \sum_{\alpha,\sigma,x'} i\, t_{\alpha,(x,y),(x',y)}\ \psi^*_{\alpha,\sigma,\tau,x,y} \psi_{\alpha,\sigma,\tau,x',y} + \text{h.c.}. \tag{1}$$

The static current-current correlator in momentum space is defined as

$$\Lambda^{(\text{xx})}(\boldsymbol{q}) = \Delta\tau \sum_{\tau,x,y} e^{-i\boldsymbol{q}\cdot\boldsymbol{r}} \left\langle j^{(\hat{x})}_{\tau,x,y} j^{(\hat{x})}_{0,0,0} \right\rangle, \tag{2}$$

where we understand $\boldsymbol{r} = (x, y)$ as usual.

The superfluid density in the thermodynamic limit is then given by

$$\rho_\text{s} = \frac{1}{4}\left[\lim_{q_x\to 0} \Lambda^{(\text{xx})}(q_x, 0) - \lim_{q_y\to 0} \Lambda^{(\text{xx})}(0, q_y)\right]. \tag{3}$$

For a finite L, we use

$$\rho_\text{s}^{(L)} = \frac{1}{4}\left[\Lambda^{(\text{xx})}\left(\frac{2\pi}{L}, 0\right) - \Lambda^{(\text{xx})}\left(0, \frac{2\pi}{L}\right)\right], \tag{4}$$

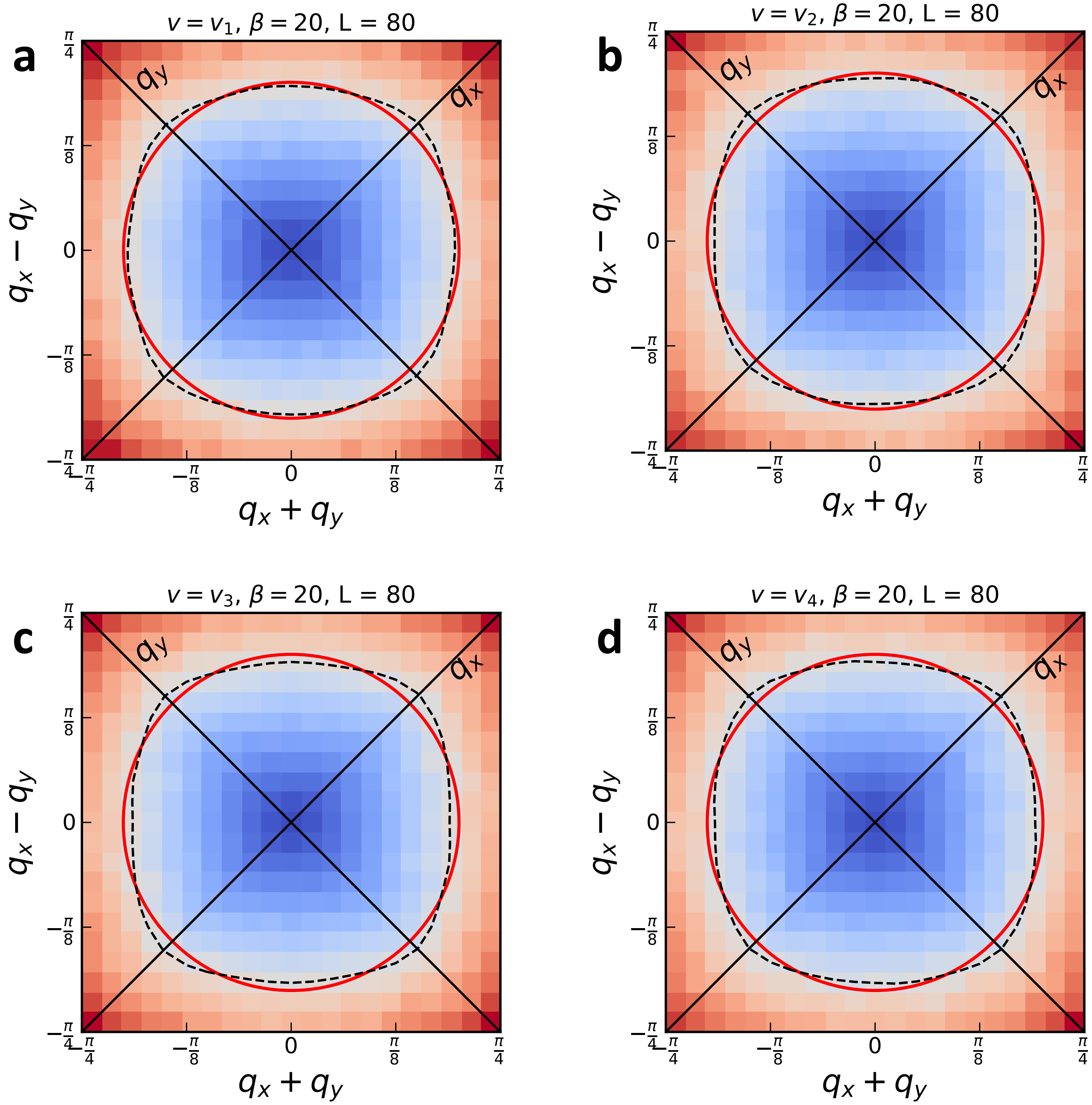

Supplementary Fig. 3: **Density plots of static spin susceptibility at criticality.** Zoomed-in plot of $\chi^{-1}(\boldsymbol{q})$ for v_i, $i = 1, 2, 3, 4$ at $\beta = 20, L = 80$, $r = r_c$. The color bar is absent for the reason explained in the caption of Fig. 7.

as a finite-size estimate of ρ_s. In practice, $\rho_\mathrm{s}^{(L)}$ depends weakly on L, as is seen by others [2]. For all nesting parameters, at the lowest temperature ($\beta = 80$), we measure $\rho_\mathrm{s}^{(L)} \approx 0$, indicating the lack of superconductivity at the parameters values we study.

SUPPLEMENTARY NOTE 3. CALCULATION OF FERMIONIC OBSERVABLES

Here we expound upon the computation of the fermion Green's function of Eq. (35) and the superfluid density of Supplementary Eq. (2) using the techniques outlined in the Methods Section.

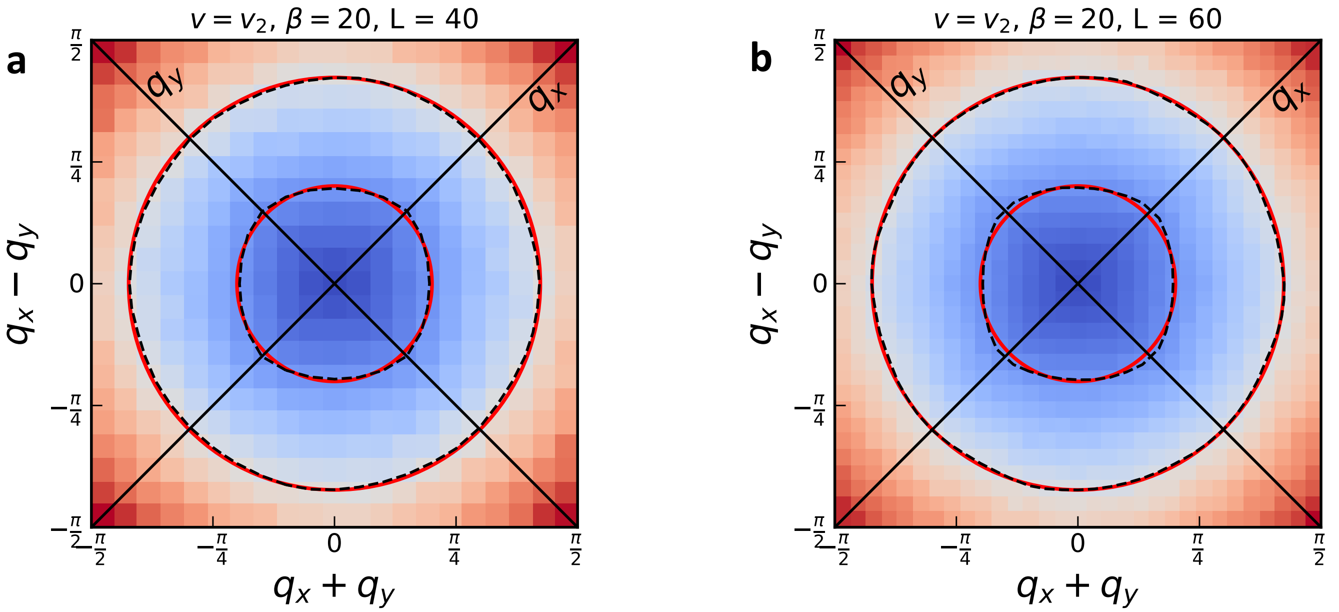

Supplementary Fig. 4: **Density plots of static spin susceptibility at criticality at smaller L.** Partially zoomed-in plot of $\chi^{-1}(\boldsymbol{q})$ for $v = v_2, r = r_c, \beta = 20$ and (a) $L = 40$ and (b) $L = 60$. The red circles are drawn at the same radii for all system sizes. Compare to Fig. 7b. The color bar is absent for the reason explained in the caption of Fig. 7.

In order to recover the Green's function as an expected diagonal of the form of Eq. (36) we simply take

$$O_\phi = \widetilde{\mathcal{F}}^\dagger \, D(\phi)^{-1} \, \widetilde{\mathcal{F}}, \tag{5}$$

where $\widetilde{\mathcal{F}}$ is the twisted discrete Fourier transform defined in the Methods Section. Note that by rewriting

$$D(\phi)^{-1} = D(\phi)^\dagger \left[D(\phi)D(\phi)^\dagger\right]^{-1} \tag{6}$$

we can perform a matrix-vector multiplications by O_ϕ by performing linear solves of the form of Eq. (22), together with fast Fourier operations and sparse matrix-vector multiplications.

It can be shown via Wick's theorem together with laborious algebraic manipulations that

$$\begin{aligned}\Lambda^{(\mathrm{xx})}(\boldsymbol{q}) \quad = \quad & \Big\langle \left[\mathrm{diag}(AG^0_\phi BAG^0_\phi)\cdot\mathbf{1}\right] - \left[\mathrm{diag}(AG^0_\phi)\cdot\mathrm{diag}(B)\right]\left[\mathrm{diag}(AG^0_\phi)\cdot\mathbf{1}\right] \\ & + \left[\mathrm{diag}(G^0_\phi A^\dagger BG^0_\phi A^\dagger)\cdot\mathbf{1}\right] - \left[\mathrm{diag}(G^0_\phi A^\dagger)\cdot\mathrm{diag}(B)\right]\left[\mathrm{diag}(G^0_\phi A^\dagger)\cdot\mathbf{1}\right] \\ & - \left[\mathrm{diag}(AG^0_\phi A^\dagger BG^0_\phi)\cdot\mathbf{1}\right] + \left[\mathrm{diag}(G^0_\phi A^\dagger)\cdot\mathrm{diag}(B)\right]\left[\mathrm{diag}(AG^0_\phi)\cdot\mathbf{1}\right] \\ & - \left[\mathrm{diag}(G^0_\phi BAG^0_\phi A^\dagger)\cdot\mathbf{1}\right] + \left[\mathrm{diag}(AG^0_\phi)\cdot\mathrm{diag}(B)\right]\left[\mathrm{diag}(G^0_\phi A^\dagger)\cdot\mathbf{1}\right] \Big\rangle,\end{aligned} \tag{7}$$

where $G^0_\phi := D(\phi)^{-1}$, and the diagonal matrices A and B are defined by

$$A_{(\alpha,\sigma,\tau,x,y),(\alpha',\sigma',\tau',x',y')} = \delta_{\alpha\alpha'}\delta_{\sigma\sigma'}\delta_{\tau\tau'}\delta_{yy'}\; t_{\alpha,(x,y),(x',y')} \tag{8}$$

and

$$\begin{aligned} & B_{(\alpha,\sigma,\tau,x,y),(\alpha',\sigma',\tau',x',y')} = \\ & \frac{\Delta\tau}{N_\tau L^2}\;\delta_{\alpha\alpha'}\delta_{\sigma\sigma'}\delta_{\tau\tau'}\delta_{xx'}\delta_{yy'}\; e^{-i\boldsymbol{q}\cdot(x,y)}.\end{aligned} \tag{9}$$

Observe that $B = B_{\boldsymbol{q}}$ implicitly depends on the choice $\boldsymbol{q}$, but for any specific choice of $\boldsymbol{q}$, the angle-bracketed expression in Supplementary Eq. (7) can be obtained in computational time dominated by the cost of a constant number of linear solves of the form of Eq. (22). Note that for any term in which diagonals appear within a product, independent diagonal estimators of the form of Eq. (38) must be used for each in order to obtain a consistent estimator for the expression in Supplementary Eq. (7).

SUPPLEMENTARY NOTE 4. ORDER OF THE TRANSITION

Although the effective action of Eq. (1) is modeling a continuous phase transition, the transition can be first-order, especially for $u = 0$ [3]. In order to check that the transition is indeed second-order for all the parameter values we use in the main text, we plot the inverse spin susceptibility $\chi^{-1} = \chi^{-1}(0, \mathbf{0})$ as a function of r for $v = v_1$, $\beta = 40, L = 20$. We can see that even at low temperatures, the susceptibility turns on in a continuous fashion. This is in contrast to

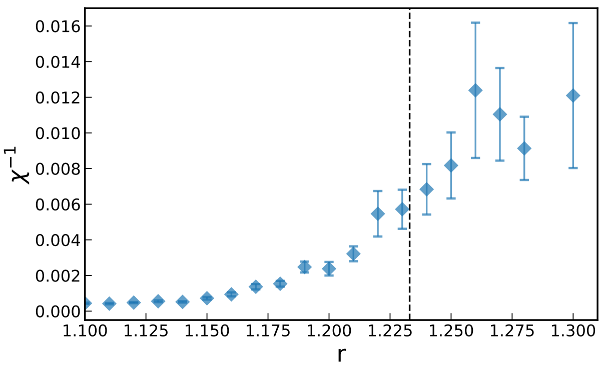

Supplementary Fig. 5: **The (unnormalized) inverse susceptibility.** χ^{-1} for $v = v_1$, $\beta = 40, L = 20$ as a function of r. The error bars denote the one sigma confidence interval.

the first-order behavior observed in Ref. [4] for certain parameter values, where χ^{-1} develops a pronounced jump at lower temperatures.

SUPPLEMENTARY NOTE 5. PRECONDITIONER PERFORMANCE

In this section we numerically validate our choice of preconditioner as described in the Methods Section. All experiments are performed for our standard choices of model parameters $u = 0.0$, $g = 0.7\sqrt{2}$, $c = 3.0$, as well as the choice $v = v_2 \approx 0.072$ for the nesting parameter and the critical parameter value $r = 1.26$.

In Supplementary Fig. 6, we fix $N_\tau = 100$ and plot the average speedup provided by the preconditioner over an HMC run, measured both in terms of wall clock time and CG iterations.

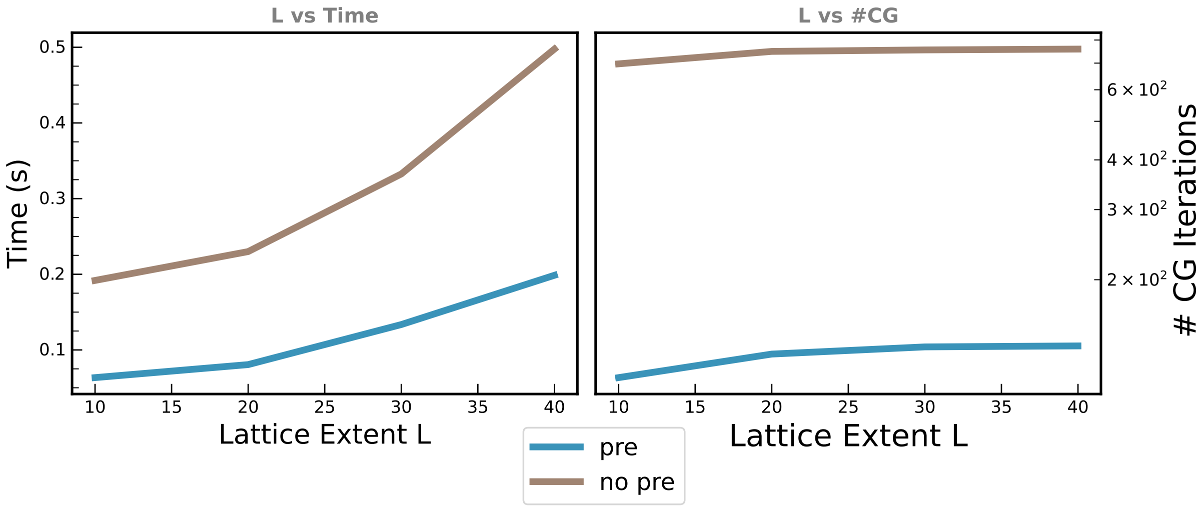

Supplementary Fig. 6: **Preconditioner performance.** Average preconditioner speedup over an HMC run as a function of lattice extent L, where $N_\tau = 100$ is fixed, measured both in terms of wall clock time and number of CG iterations.

To bolster the practical conclusions of Supplementary Fig. 6 and see more concretely how the preconditioner affects the conditioning of the linear solve, we plot in Supplementary Fig. 7 a histogram of the condition numbers of the matrices $M = DD^\dagger$ and $M = PDD^\dagger$, where $D = D(\phi)$ is the fermion matrix and P is the preconditioner of Eq. (26), collected over configurations ϕ obtained from an HMC run, for two alternative choices $N_\tau = 50, 100$.

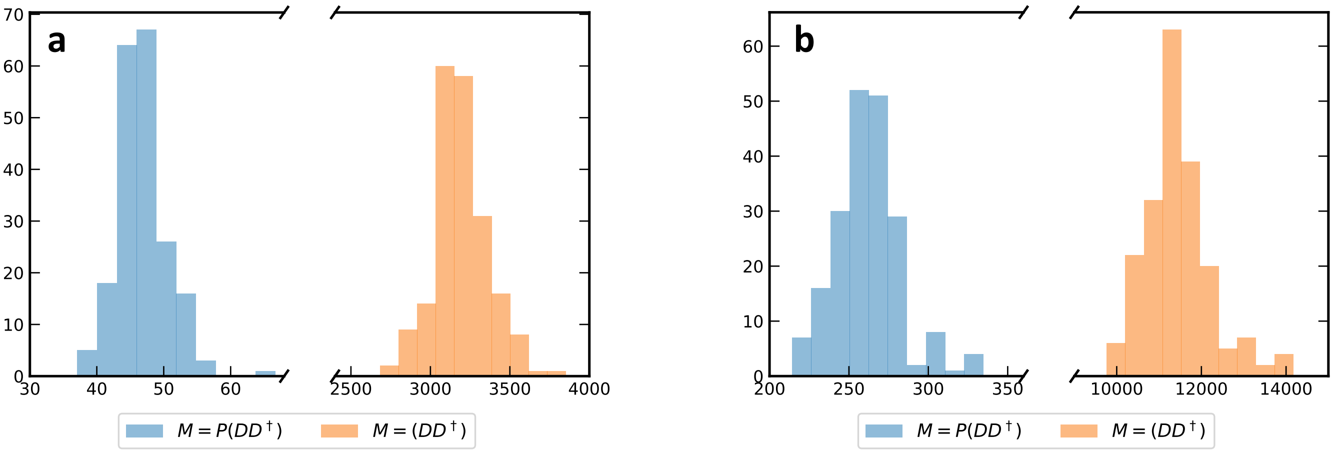

Supplementary Fig. 7: **Effect of preconditioner on condition numbers.** Histogram of condition numbers of $M = DD^\dagger, PDD^\dagger$, where $D = D(\phi)$ is the fermion matrix and P is the preconditioner, based on samples ϕ collected from an HMC run. (a) $N_\tau = 50$. (b) $N_\tau = 100$.

Finally, in Supplementary Fig. 8 we validate the claim from Methods Section that the advantage of the preconditioner becomes more significant in the limit of small Trotter step Δ_τ. Although in the rest of this work we only consider $\Delta_\tau = 0.1$, this limit nonetheless provides one way of understanding the advantage of the preconditioner besides the motivation via exactness for a the choice of zero coupling $g = 0$.

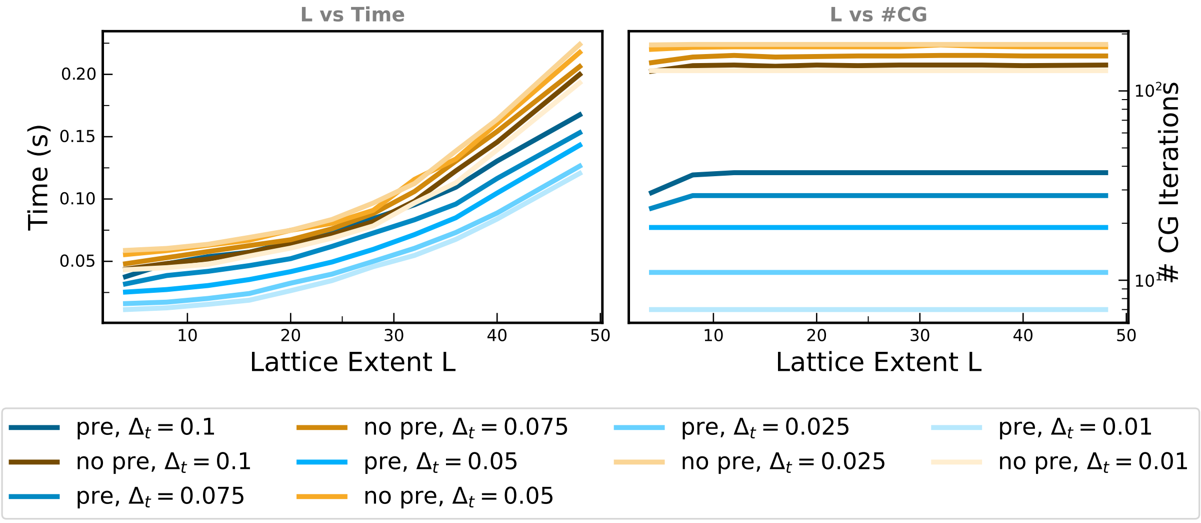

Supplementary Fig. 8: **Trotter steps and preconditioner performance.** Average preconditioner speedup over an HMC run as a function of lattice extent L, where $N_\tau = 50$ is fixed, measured both in terms of wall clock time and number of CG iterations. Lines correspond to various choices of Trotter step size Δ_τ with and without precondtioning.

SUPPLEMENTARY NOTE 6. NUMERICAL PERFORMANCE DETAILS

Here we provide more details on the numerical performance of our algorithm, expounding on the summary main in the the Methods Section. As a concrete observable, we track the growth of the integrated autocorrelation time τ_{int} of

the total SDW susceptibility $\chi \equiv \chi(0, \mathbf{0})$ at criticality.

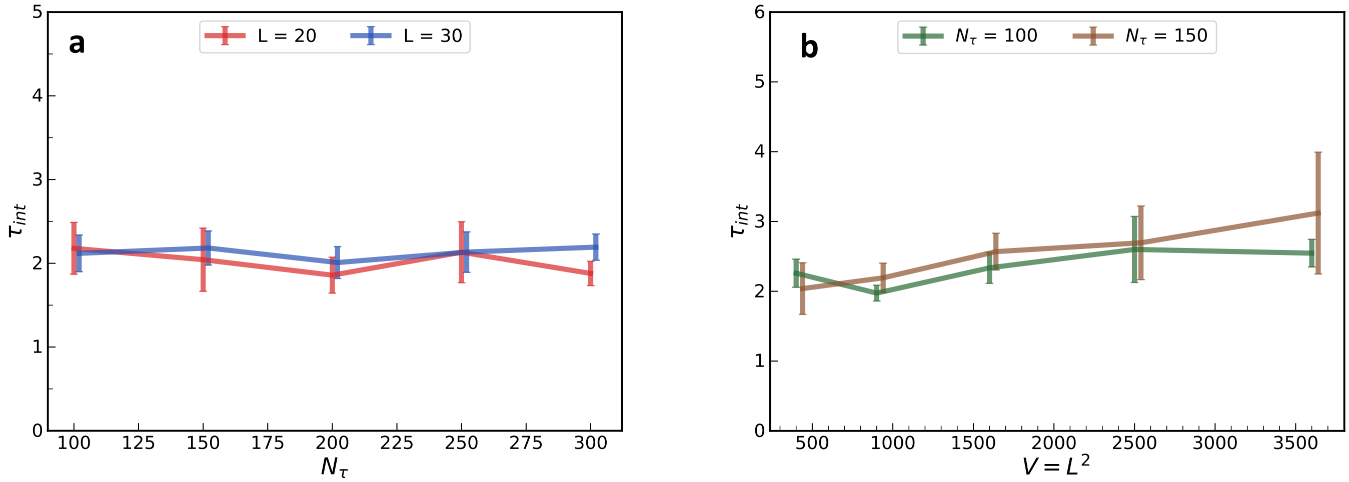

Supplementary Fig. 9: **Scaling of autocorrelation time.** Integrated autocorrelation time τ_{int} for the SDW susceptibility χ under the auto-tuned HMC algorithm. Each value is computed from the average of five Markov chains, and the error bars are the corresponding one sigma deviation. (a) Plot against N_τ for $L = 20$ and $L = 30$. (b) Plot against L for $N_\tau = 100$ and $N_\tau = 150$. In both cases, we observe evidence of constant scaling of τ_{int} with respect to system size.

To test the performance, we ran the HMC algorithm in two studies: one in which the lattice volume is fixed to sizes $L = 20, 30$ and inverse temperature scaled from $N_\tau = 100$ to $N_\tau = 300$, and one in which the number of imaginary time steps is fixed to $N_\tau = 100, 150$ while lattice extent L is scaled from 20 to 60. For each scenario, empirical averages and error bars were computed using 5 Markov chains. In Supplementary Fig. 9, we see that our algorithm exhibits constant scaling of τ_{int} with respect to both lattice volume and inverse temperature.

The number of leapfrog integration steps per effective sample is plotted in Supplementary Fig. 10. This number roughly tracks the total algorithmic cost in terms of the number of linear solves of the form Eq. (22). A power law fit $y = ax^b$ is applied to the measurements to extract the scaling exponents z_1, z_2. With respect to N_τ, we observe that $z_1 = b - 1/4 \approx 0.5$. With respect to V, we observe that $z_2 = b - 1/4 \approx 0$. As noted in the main text, this implies an absence of critical slowing down with respect to the lattice volume V for the auto-tuned HMC algorithm presented in this work.

Finally, in Supplementary Fig. 11, we show the wall clock time per effective sample with respect to V and N_τ, where we can see that the scaling is roughly linear with respect to V and superlinear with respect to N_τ.

[1] Scalapino, D. J., White, S. R. & Zhang, S. Insulator, metal, or superconductor: The criteria. *Phys. Rev. B* **47**, 7995–8007 (1993). URL https://link.aps.org/doi/10.1103/PhysRevB.47.7995.

[2] Schattner, Y., Gerlach, M. H., Trebst, S. & Berg, E. Competing orders in a nearly antiferromagnetic metal. *Phys. Rev. Lett.* **117**, 097002 (2016). URL https://link.aps.org/doi/10.1103/PhysRevLett.117.097002.

[3] Altshuler, B. L., Ioffe, L. B. & Millis, A. J. Low-energy properties of fermions with singular interactions. *Phys. Rev. B* **50**, 14048–14064 (1994). URL http://link.aps.org/doi/10.1103/PhysRevB.50.14048.

[4] Klug, M. J., Scheurer, M. S. & Schmalian, J. Hierarchy of information scrambling, thermalization, and hydrodynamic flow in graphene. *Phys. Rev. B* **98**, 045102 (2018). URL https://link.aps.org/doi/10.1103/PhysRevB.98.045102.

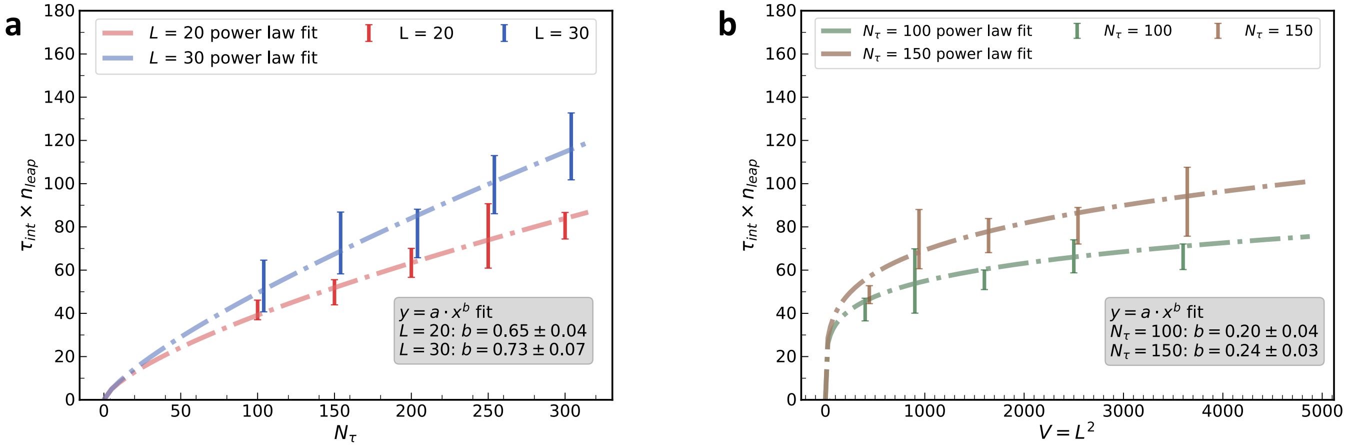

Supplementary Fig. 10: **Scaling of computational cost.** Benchmarks of integration steps per effective sample, $\tau_{\rm int} \times n_{\rm leap}$, for the SDW susceptibility χ. Each value is computed from the average of five Markov chains, and the error bars are the corresponding one sigma deviation. (a) Plot against N_τ for $L = 20$ and $L = 30$. (b) Plot against $V = L^2$ for $N_\tau = 100$ and $N_\tau = 150$. In both cases, a power-law fit is shown.

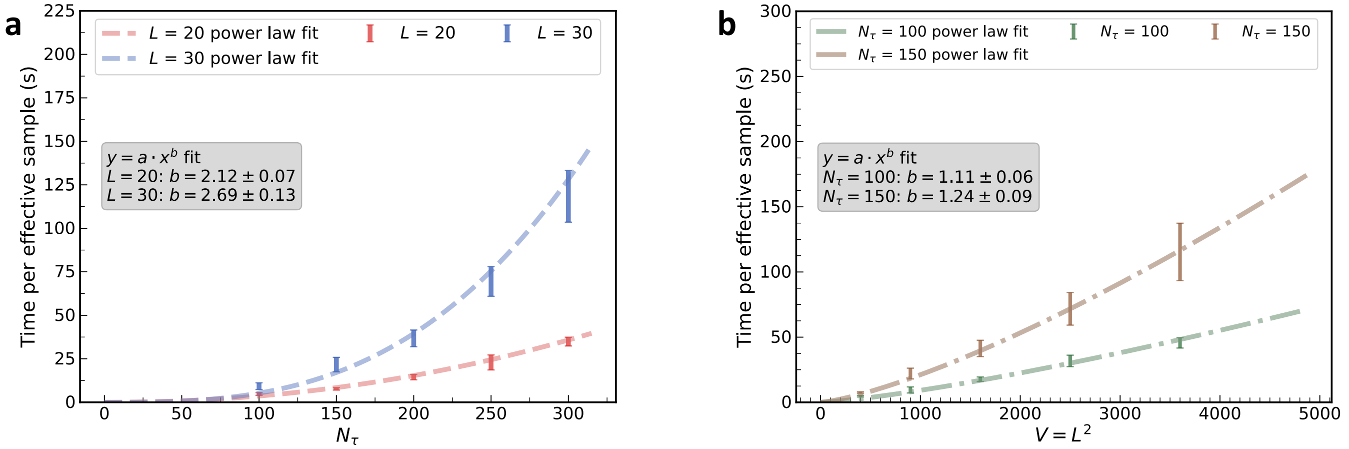

Supplementary Fig. 11: **Scaling of wallclock time.** Benchmarks of wallclock time per effective sample under the auto-tuned HMC algorithm. Each value is computed from the average of five Markov chains, and the error bars are the corresponding one sigma deviation. (a) Times scaled across N_τ for L values of $L = 20$ and $L = 30$. (b) Times scaled across $V = L^2$ for N_τ values of $N_\tau = 100$ and $N_\tau = 150$. In both cases, a power-law fit is shown.
