# Supplementary material for: Non-Hertz-Millis scaling of the antiferromagnetic quantum critical metal via scalable Hybrid Monte Carlo
Source: Nat Commun. 2023 May 3;14:2547. doi: 10.1038/s41467-023-37686-4 (PMC10156689; doi:10.1038/s41467-023-37686-4)
Supplement: Supplementary file 2 — Peer Review File [file 41467_2023_37686_MOESM2_ESM.pdf]

nature portfolio

Peer Review File

Non-Hertz-Millis scaling of the antiferromagnetic quantum critical metal via scalable Hybrid Monte Carlo

Open Access This file is licensed under a Creative Commons Attribution 4.0 International License, which permits use, sharing, adaptation, distribution and reproduction in any medium or format, as long as you give appropriate credit to the original author(s) and the source, provide a link to the Creative Commons license, and indicate if changes were made. In the cases where the authors are anonymous, such as is the case for the reports of anonymous peer reviewers, author attribution should be to 'Anonymous Referee' followed by a clear attribution to the source work. The images or other third party material in this file are included in the article's Creative Commons license, unless indicated otherwise in a credit line to the material. If material is not included in the article's Creative Commons license and your intended use is not permitted by statutory regulation or exceeds the permitted use, you will need to obtain permission directly from the copyright holder. To view a copy of this license, visit http://creativecommons.org/licenses/by/4.0/.

REVIEWER COMMENTS

Reviewer #1 (Remarks to the Author):

Dear Authors and Editors,

The Authors of the paper "Non-Hertz-Millis scaling of the antiferromagnetic quantum critical metal via scalable Hybrid Monte Carlo" study an O(3) spin-fermion model numerically via the Hamiltonian Monte Carlo(HMC) method and support their results with RG results. They numerically extract spin susceptibilities and observe a violation of the assumed Hertz-Millis predictions that is in support of their earlier work [12].

Their method of choice is HMC, a method that has been shown to be very well suited for electron-phonon models, e.g. their refs [35,42] and is hence suited to simulate the large system sizes required for their question in a numerically unbiased manner while still providing results that go beyond previous results using the BSS method [12].

Validity:

The numerically shown data seems robust and to support their claim. I think it would be very beneficial If the authors could amend figures 5 and 6 and the conclusion drawn from it with a discussion of the errors. Parts of it are already there since the extraction of the exponents is explained, but ideally one would like to have an estimate on the errors on the exponents.

Data and Methodology:

As already noted HMC is a very well-suited tool for this approach.

Potential pitfalls like ergodicity have been discussed and solved for this particular model and suitable algorithms for the hyperparameter tuning have been gathered from the literature and compiled in a separate section.

I consider the separate discussions on the preconditioners for HMC and the choice and estimation of the mass matrix valuable contributions.

Clarity and content:

The Text is very well-written and especially the compiled results for HMC can prove useful for future works in sold-state physics.

Suggested Improvements:

- As already noted Fig.5 + Fig.6 and the conclusions drawn from it would benefit from an elaborated discussion of the error.

- Section IV.C.1 The choice of their preconditioner reminds me of the U=0 cases in HMC on Hubbard type models(ref [33]). It has been observed that it works reasonably well for non-interacting models but going away from that point usually leads to detoriation. For the Hubbard model there is a plethora of works detailing this available in this book chapter: https://web.cs.ucdavis.edu/~bai/publications/bcsy09.pdf

Hence I strongly believe the authors' preconditioner is not as general as the authors would like it to be....

Also showing the spectra of an unpreconditioned matrix vs a preconditioned matrix would support this section.

- Section IV.E.2 is a valuable contribution to HMC in solid state physics. I think it would be beneficial to point out that the energy difference is directly related to the acceptance rate with relevant papers being https://doi.org/10.1016/S0550-3213(01)00129-8 or more recently https://arxiv.org/pdf/1912.03253.pdf

- Eq. 37: brackets would maybe make the order of operations clearer.

Additionally there are a couple of typos:

pg. 8: "...that that the actual leading order..."

pg. 10: "... scaling with with respect to L..."

pg. 14 "... antifferomagnetic..."

pg. 16: "... other sing-problem-free ..."

pg. Appendix pg. 4: "We can see that that even at low ..."

Verdict:

The paper is strong and I support publication in Nature communications when above comments are clarified.

Reviewer #2 (Remarks to the Author):

In this work the authors develop and use an efficient Hybrid Monte Carlo method to study the physics of a quantum critical point involving fermions interacting near a Fermi surface. The physics studied is highly non-perturbative and the numerical method is one of the only ab-initio methods to access the physics. While this problem has been studied before using other numerical methods, the earlier studies were able to access only small lattices. The main conclusions of the current work is that conclusions of the previous studies, which had found that the scaling relation suggested by Hertz and Millis is valid, are in fact incorrect. The reason for this is apparently the small lattice sizes used in the previous studies. By going to larger systems sizes, the authors claim to see the expected non-Hertz Millis scaling.

The quantum critical point that is studied in this work separates a metal from an anti-ferromagnet. The physics arises due to gapless fermions close to the Fermi surface interacting strongly to create an anti-ferromagnet with gapped fermions. While the full microscopic theory is difficult to study, from what I understand, the authors study a low energy effective theory that captures the same physics. This theory is given in Eq.(1). One important point to note is that, instead of starting from a theory where fermions interact via some effective Coulomb potential, as is more natural in a real material, the authors study a Yukawa type theory where the physics of the anit-ferromagnetic fluctuations are already introduced by hand through a bosonic field. The physics of the Fermi surface and the special points (hot spots) where the anti-ferromagnetic order begins to form are described in Fig. 1. In order write down a more complete theory the authors modify the theory in Eq.(1) and study Eq.(2), which is free of sign problems. Thus, the authors design a Hamiltonian which can capture the physics of the quantum critical point.

Both theories Eq.(1) and (2) have been proposed earlier. In particular Eq.(1) was used in some parameter regime to perform a controlled study in Ref. [12] as the authors explain in II C and Eq.(2) was proposed as a way to perform Monte Carlo calculations in Ref. [16]. The special role of the hot spots in the physics enters through the nesting angle, which enters the model (Eq.(2)) via the hopping terms. In this work the authors study five different values of the nesting parameters which means five different values of hopping parameters given in Eqs. (3-7).

In section III the authors discuss the results. As I understand, in addition to the scaling plots Fig. 5 and Fig. 8, which qualitatively agree with the scaling predictions of Ref. [12], the main results are also the dynamical exponent z and the anomalous dimension eta, which depend on the nesting parameter. These are plotted in Fig 6. Unfotunately, these values do not seem to agree with the predictions of [12] given in Eqs. (9) and (10) especially close to v=0 where the analysis of [12] is expected to be correct. The authors point out a possible reason for this. But, I am worried that there may be more to the story here that the authors point out.

I believe that a physical system with a dynamical exponent z enclosed in a box of size L, will be at low temperatures only when beta \approx L^z. Also, I would argue that the finite T scalings given in Eq. (12) would be valid only at sufficiently low temperatures close to the critical point. Assuming these two approximate assumptions, and the fact that the authors find 1.6 < z < 2.0, a good consistency check would be study beta in the range 120-400 on a (20 x 20) lattice, and beta = 1000 - 6400 at L = 80. I can understand that these extreme values of beta are most likely beyond the scope of the current studies, but perhaps not impossible to achieve in the future. But from the perspective of discrepancy at small v, isn't it likely that one enters a different regime of scaling at these truly low temperatures? I believe a discussion is necessary to explain why beta = 20 on an 80 x 80 lattice is still low enough temperature to capture the quantum critical regime. I am more comfortable to agree that beta = 80 on a 20 x 20 lattice is reasonably small. In fact, the data is fluctuating much more in Fig 8a (beta = 20 L = 80) as compared to Fig 8b (beta = 80, L=20) as I would have expected. I believe a more careful discussion about this point is warrented.

In terms of the algorithm itself, the authors seem to have developed a fine tuned algorithm which may be useful for other studies. Can the authors discuss more about the condition number of the preconditioned matrix (discussed in section IV C behaves? For example what guarantees that there are no exact zero modes of D? Can the authors plot the low lying eigenvalue distribution of D in a typical background field as a function of Nt for a fixed L? I would be curious to understand how the low lying eigenvalues change at very low temperatures and understand the physics of L.

Also is it possible that there are topological configurations of the bosonic field, which divide the space of fields into classes with exact zero modes separating them. If true one might worry that the algorithm could be stuck in one of these sectors? Such problems naturally arise in the studies of Quantum Chromodynamics and is an important bottleneck.

Overall I found the paper stimulating and I recommend it be published after the authors have addressed the comments/concerns I have raised above.

In addition I noticed a few minor issues while reading.

1. I may have missed the discussion, but what is the motivation to choose the values for u = 0, g = 1, c = 3 and the nesting parameters discussed in II B?

2. In Fig 8. the authors discuss two different scaling plots at different beta and L. I presume beta = 1/T? I could not see this stated, but may be I missed.

3. The authors say at one point that "the critical scaling at the SDW QCP is governed by the theory of Ref. [12] for all the nesting values we study", while immediately in the next paragraph they say "There are two parts of the data that are seemingly in contrast to the predictions of Ref. [12]." Perhaps there is a more consistent way to phrase these two contradictory statements.

Reviewer #3 (Remarks to the Author):

The authors numerically study the O(3) spin-fermion model. Using a Hybrid Monte Carlo study at 80x80 sites, they extract the scaling exponents and functional form of the spin susceptibility. From the numerical calculation, they found a violation of Hertz-Millis forms. This result seems to be consistent with a previous analytic study by Schlief, Lunts, and Lee. Their work provides concrete predictions which can be observed by neutron scattering experiments. The paper is recommended for publication after addressing the comments below.

The comments and questions are as follows:

1) The authors claimed that their functional form of the susceptibility (Eq.15) is consistent with the form in Ref.12. I think that it will be in Appendix D in Ref.12. However, with just the given manuscript, it is hard to convince the claim. Could you provide the functional form which you used in the analysis? I think that the author should present the form they used in the main text, or appendix (supplemental materials), to be self-contained.

2) The authors also claimed that their study shows the `strong' violation of Hertz-Millis forms. In the manuscript, it seems that z may be less than 2, but not far away from 2. The authors also discussed that issue, but it seems difficult to argue that there is a 'strong' violation. Could you explain why you claim 'strong' violation, not just violation?

3) All the results are scattered in the entire manuscript, so it seems that the reader may be hard to follow up on their results. It would be better to understand if the authors add some table summarizing their numerical result and analytic expectation (from Ref.12), including the functional form of the susceptibilities, fitting z and \Delta values, etc, in the main text, not in the appendix or supplemental materials.

Response: We thank all the referees for their feedback. We have replied to all the points brought up by them. All corrections/changes in the text are written in magenta.
Additionally, we have made one small change to Fig. 9 by adding a figure, and moving two of the previous figures there to Appendix A.
Also, we have slightly altered the abstract.

REVIEWER COMMENTS

Reviewer #1 (Remarks to the Author):

Dear Authors and Editors,
The Authors of the paper "Non-Hertz-Millis scaling of the antiferromagnetic quantum critical metal via scalable Hybrid Monte Carlo" study an O(3) spin-fermion model numerically via the Hamiltonian Monte Carlo(HMC) method and support their results with RG results. They numerically extract spin susceptibilities and observe a violation of the assumed Hertz-Millis predictions that is in support of their earlier work [12].
Their method of choice is HMC, a method that has been shown to be very well suited for electron-phonon models, e.g. their refs [35,42] and is hence suited to simulate the large system sizes required for their question in a numerically unbiased manner while still providing results that go beyond previous results using the BSS method [12].

Validity:
The numerically shown data seems robust and to support their claim. I think it would be very beneficial If the authors could amend figures 5 and 6 and the conclusion drawn from it with a discussion of the errors. Parts of it are already there since the extraction of the exponents is explained, but ideally one would like to have an estimate on the errors on the exponents.

Data and Methodology:
As already noted HMC is a very well-suited tool for this approach.
Potential pitfalls like ergodicity have been discussed and solved for this particular model and suitable algorithms for the hyperparameter tuning have been gathered from the literature and compiled in a separate section.
I consider the separate discussions on the preconditioners for HMC and the choice and estimation of the mass matrix valuable contributions.

Clarity and content:
The Text is very well-written and especially the compiled results for HMC can prove useful for future works in sold-state physics.

Response: We thank the referee for their positive feedback.

Suggested Improvements:
- As already noted Fig.5 + Fig.6 and the conclusions drawn from it would benefit from an elaborated discussion of the error.

Response: This is an important point . We have added error bars to the critical exponents, and explained how we obtain them in Appendix A2.

- Section IV.C.1 The choice of their preconditioner reminds me of the U=0 cases in HMC on Hubbard type models(ref [33]). It has been observed that it works reasonably well for non-interacting models but going away from that point usually leads to detoriation. For the Hubbard model there is a plethora of works detailing this available in this book chapter: https://web.cs.ucdavis.edu/~bai/publications/bcsy09.pdf
Hence I strongly believe the authors' preconditioner is not as general as the authors would like it to be....
Also showing the spectra of an unpreconditioned matrix vs a preconditioned matrix would support this section.

- **Response:** We completely agree with the reviewer that the preconditioner is not necessarily satisfactory for large coupling and have added a comment to this effect in Section IV.C.1, together with a pointer to the reference above mentioned. Our discussion in the “Algorithmic Improvements” section, suggesting more sophisticated approaches for the linear solve to be considered in further work, was meant to address this gap, and we have clarified this point there as well.
- For the couplings studied in this paper, we do nonetheless observe improvement due to the preconditioner. Due to the large cost of plotting the entire spectrum, we instead include some numerical experiments comparing the iteration count of preconditioned vs. unpreconditioned CG at the parameters in the paper, in Appendix E.
- Note also that the preconditioner achieves superior scaling compared to the unpreconditioned alternative in the limit of small imaginary time step, as commented at the end of Section IV.C.1, as we corroborate in Appendix E. In further work, if the Trotter error due to finite time step is a concern, this may be a valuable perspective.

- Section IV.E.2 is a valuable contribution to HMC in solid state physics. I think it would be beneficial to point out that the energy difference is directly related to the acceptance rate with relevant papers being https://doi.org/10.1016/S0550-3213(01)00129-8 or more recently https://arxiv.org/pdf/1912.03253.pdf

Response: We thank the reviewer for their comment and have included this point and corresponding citations in Section IV.E.2.

- Eq. 37: brackets would maybe make the order of operations clearer.
Additionally there are a couple of typos:
pg. 8: "...that that the actual leading order..."
pg. 10: "... scaling with with respect to L..."
pg. 14 "... antifferomagnetic..."
pg. 16: "... other sing-problem-free ..."
pg. Appendix pg. 4: "We can see that that even at low ..."

Response: We thank the referee for catching these. We have fixed them.

Verdict:

The paper is strong and I support publication in Nature communications when above comments are clarified.

Reviewer #2 (Remarks to the Author):

In this work the authors develop and use an efficient Hybrid Monte Carlo method to study the physics of a quantum critical point involving fermions interacting near a Fermi surface. The physics studied is highly non-perturbative and the numerical method is one of the only ab-initio methods to access the physics. While this problem has been studied before using other numerical methods, the earlier studies were able to access only small lattices. The main conclusions of the current work is that conclusions of the previous studies, which had found that the scaling relation suggested by Hertz and Millis is valid, are in fact incorrect. The reason for this is apparently the small lattice sizes used in the previous studies. By going to larger systems sizes, the authors claim to see the expected non-Hertz Millis scaling.

The quantum critical point that is studied in this work separates a metal from an anti-ferromagnet. The physics arises due to gapless fermions close to the Fermi surface interacting strongly to create an anti-ferromagnet with gapped fermions. While the full microscopic theory is difficult to study, from what I understand, the authors study a low energy effective theory that captures the same physics. This theory is given in Eq.(1). One important point to note is that, instead of starting from a theory where fermions interact via some effective Coulomb potential, as is more natural in a real material, the authors study a Yukawa type theory where the physics of the anit-ferromagnetic fluctuations are already introduced by hand through a bosonic field. The physics of the Fermi surface and the special points (hot spots) where the anti-ferromagnetic order begins to form are described in Fig. 1. In order write down a more complete theory the authors modify the theory in Eq.(1) and study
Eq.(2), which is free of sign problems. Thus, the authors design a Hamiltonian which can capture the physics of the quantum critical point.

Both theories Eq.(1) and (2) have been proposed earlier. In particular Eq.(1) was used in some parameter regime to perform a controlled study in Ref. [12] as the authors explain in II C and Eq.(2) was proposed as a way to perform Monte Carlo calculations in Ref. [16]. The special role of the hot spots in the physics enters through the nesting angle, which enters the model (Eq.(2)) via the hopping terms. In this work the authors study five different values of the nesting parameters which means five different values of hopping parameters given in Eqs. (3-7).

In section III the authors discuss the results. As I understand, in addition to the scaling plots Fig. 5 and Fig. 8, which qualitatively agree with the scaling predictions of Ref. [12], the main results are also the dynamical exponent z and the anomalous dimension eta, which depend on the nesting parameter. These are plotted in Fig 6. Unfotunately, these values do not seem to agree with the predictions of [12] given in Eqs. (9) and (10) especially close to v=0 where the analysis of [12] is expected to be correct. The authors point out a possible reason for this. But, I am worried that there may be more to the story here that the authors point out.

I believe that a physical system with a dynamical exponent z enclosed in a box of size L, will be at low temperatures only when beta \approx L^z. Also, I would argue that the finite T scalings given in Eq. (12) would be valid only at sufficiently low temperatures close to the critical point. Assuming these two approximate assumptions, and the fact that the authors find 1.6 < z < 2.0, a

good consistency check would be study beta in the range 120-400 on a (20 x 20) lattice, and beta = 1000 - 6400 at L = 80. I can understand that these extreme values of beta are most likely beyond the scope of the current studies, but perhaps not impossible to achieve in the future. But from the perspective of discrepancy at small v, isn't it likely that one enters a different regime of scaling at these truly low temperatures? I believe a discussion is necessary to explain why beta = 20 on an 80 x 80 lattice is still low enough temperature to capture the quantum critical regime. I am more
comfortable to agree that beta = 80 on a 20 x 20 lattice is reasonably small. In fact, the data is fluctuating much more in Fig 8a (beta = 20 L = 80) as compared to Fig 8b (beta = 80, L=20) as I would have expected. I believe a more careful discussion about this point is warrented.

Response:

We thank the referee for bringing up this important and often confusing point. For the temperature T of a physical system to be ‘low’, the T must be smaller than all the microscopic, i.e. UV scales of the system. For example, it is important that T be much smaller than the Fermi energy E_f. In that case, T acts as an IR scale, which is usually the relevant regime for quantum effects to take place. On the other hand, the energy scale associated with finite size effects, E_L = 1/L^z, is another IR energy scale. In condensed matter systems, this scale is absurdly small, i.e. E_L ~ 10^{-23*z}, much much smaller than T, and the finite-size effects do not affect the IR physics at the scale E ~ T.
The relationship between the two IR cutoffs E_L and T is important for Eq. (12) though, as the referee points out. Eq. (12) is used to get the scaling of the susceptibility with frequency and momenta for fixed T and L (Eqs. (13-14)). These scaling behaviors will only hold above some IR scales, which are not known, but can only be functions of E_L and T. Therefore, if the critical exponents were extracted with finite-size scaling, the correct increase of beta and L would follow beta ~ L^z, as the referee notes. However, here our critical exponents are not found in such a way, as they are each extracted from a system with fixed T and L. The IR cutoffs of these scalings are then read off from Fig. 5, and it is not known which of the two IR scales defines this IR cutoff. The important point is that the existence of the scaling region in the first place proves that we are at a low enough temperature to observe the critical scaling.
This is explained in more detail below Eqs. (13) and (14). However, to clarify the point even further, and to avoid similar confusion, we have added an extra small discussion on this near the end of Section II.

In terms of the algorithm itself, the authors seem to have developed a fine tuned algorithm which may be useful for other studies. Can the authors discuss more about the condition number of the preconditioned matrix (discussed in section IV C behaves? For example what guarantees that there are no exact zero modes of D? Can the authors plot the low lying eigenvalue distribution of D in a typical background field as a function of Nt for a fixed L? I would be curious to understand how the low lying eigenvalues change at very low temperatures and understand the physics of L.

- **Response:** We have added a bit more discussion of the function of the preconditioner in Section IV.C.1 to clarify that it leaves room for improvement for large couplings. The “Algorithmic Improvements” section suggests more sophisticated approaches to the linear solve that may be preferable in this regime. We have also added experiments in Appendix E demonstrating the practical utility of our choice of preconditioner, as well as

the role in controlling poor conditioning due to the small imaginary time step size. Due to the randomness of the field, exact zero modes of D are not really a concern because they are nongeneric (hence will appear with zero probability). However, the distribution of the condition number of DD* and that of the preconditioned alternative (K^{-1}D)(K^{-1}D)* are still of interest as the reviewer points out, and we have plotted these distributions for a few values of Nt in Appendix E.

Also is it possible that there are topological configurations of the bosonic field, which divide the space of fields into classes with exact zero modes separating them. If true one might worry that the algorithm could be stuck in one of these sectors? Such problems naturally arise in the studies of Quantum Chromodynamics and is an important bottleneck.

Response: We thank the reviewer for bringing up this important point. In this theory there are no such topological sectors. This is due to the fact that our model is not a “non-linear sigma model”, and the O(3) spin is not constrained to be of unit length. We add a note on this at the end of Section V B, when we discuss theories for future studies, which include gauge theories with this issue.

Overall I found the paper stimulating and I recommend it be published after the authors have addressed the comments/concerns I have raised above.

In addition I noticed a few minor issues while reading.

1. I may have missed the discussion, but what is the motivation to choose the values for u = 0, g = 1, c = 3 and the nesting parameters discussed in II B?

Response: This is a good question. We have clarified this right below where we state our choice for those parameters at the end of Section II B.

2. In Fig 8. the authors discuss two different scaling plots at different beta and L. I presume beta = 1/T? I could not see this stated, but may be I missed.

Response: Beta is defined on page 2 in the Introduction, but we added a reminder in the third paragraph of Sec. III.

3. The authors say at one point that "the critical scaling at the SDW QCP is governed by the theory of Ref. [12] for all the nesting values we study", while immediately in the next paragraph they say "There are two parts of the data that are seemingly in contrast to the predictions of Ref. [12]." Perhaps there is a more consistent way to phrase these two contradictory statements.

Response: We thank the referee for pointing this out. We rephrased these two paragraphs so as not to confuse the reader.

Reviewer #3 (Remarks to the Author):

The authors numerically study the O(3) spin-fermion model. Using a Hybrid Monte Carlo study at 80x80 sites, they extract the scaling exponents and functional form of the spin susceptibility. From the numerical calculation, they found a violation of Hertz-Millis forms. This result seems to be consistent with a previous analytic study by Schlief, Lunts, and Lee. Their work provides

concrete predictions which can be observed by neutron scattering experiments. The paper is recommended for publication after addressing the comments below.

Response: We thank the referee for their comments and suggestions.

The comments and questions are as follows:

1) The authors claimed that their functional form of the susceptibility (Eq.15) is consistent with the form in Ref.12. I think that it will be in Appendix D in Ref.12. However, with just the given manuscript, it is hard to convince the claim. Could you provide the functional form which you used in the analysis? I think that the author should present the form they used in the main text, or appendix (supplemental materials), to be self-contained.

Response: This comment was referring to some arguments about the form of higher order corrections to the susceptibility in powers of v. We have removed this comment, since it is confusing, quite vague, and not very relevant or important for the present analysis.
Also, the form of Appendix D in Ref. 12 (Eq. (D7)) doesn't really apply here, since it is for the ultra-low energy limit, where the flow of v is taken into account as well.

2) The authors also claimed that their study shows the `strong' violation of Hertz-Millis forms. In the manuscript, it seems that z may be less than 2, but not far away from 2. The authors also discussed that issue, but it seems difficult to argue that there is a 'strong' violation. Could you explain why you claim 'strong' violation, not just violation?

Response: For the smallest value of v, we find z \approx 1.66, which is about an 18% change from z = 2. Of course this is a matter of perspective, but we see this change as a `large' one. Also, the other main deviation we find is a spatial symmetry breaking of the functional form. Putting these two pieces together, we feel that characterizing the deviation as 'strong' is justified.

3) All the results are scattered in the entire manuscript, so it seems that the reader may be hard to follow up on their results. It would be better to understand if the authors add some table summarizing their numerical result and analytic expectation (from Ref.12), including the functional form of the susceptibilities, fitting z and \Delta values, etc, in the main text, not in the appendix or supplemental materials.

Response: This is a great suggestion. We have added a table to the introduction that summarizes our findings and their (in)consistency with Hertz-Millis theory and Ref. 12.

REVIEWERS' COMMENTS

Reviewer #1 (Remarks to the Author):

All raised points have been satisfactorily addressed.

Reviewer #2 (Remarks to the Author):

I thank the authors for considering my comments and addressing my concerns. I feel they have adequately addressed my concerns. I recommend the paper with the revisions.

Reviewer #3 (Remarks to the Author):

The authors have answered sincerely all my questions and suggestions and revised their manuscript. The manuscript looks good now and I have no additional comments to make.
